# A Factorial Model of the Minimum Metabolic Demand for Protein and Indispensable Amino Acids in Young Adult Males: Implications for Current Recommendations

**DOI:** 10.1016/j.tjnut.2026.101417

**Published:** 2026-02-12

**Authors:** Carlene S Starck, Robert R Wolfe, Paul J Moughan

**Affiliations:** 1 Riddet Institute, Massey University, Palmerston North, New Zealand; 2 Starck Science, Precision Nutrition Research and Innovation, Waikato, New Zealand; 3 Department of Geriatrics, Donald W. Reynolds Institute on Aging, Center for Translational Research in Aging & Longevity, University of Arkansas for Medical Sciences, Little Rock, AR, United States

**Keywords:** factorial model, protein requirements, indispensable amino acid requirements, obligatory amino acid losses, dietary protein

## Abstract

**Background:**

Consensus concerning human dietary protein and indispensable amino acid (IAA) recommendations is lacking. A factorial model capable of predicting the minimum metabolic demand (MMD) for protein and each IAA and elucidating underlying processes would provide valuable mechanistic insight.

**Objectives:**

The study aimed to provide an understanding of the basal metabolic demand for protein and each IAA in the adult human using a factorial approach.

**Methods:**

A factorial model for the MMD for each AA and protein was developed by summing obligatory AA losses. Oxidative and gut endogenous losses have been given previously. Urinary free amino acids and peptides, hair, skin, nail, and miscellaneous losses, and losses because of functional roles and irreversible modification were estimated from the literature. MMD values were compared with current Food and Agriculture Organization/World Health Organization recommendations and used as reference patterns to calculate protein quality by the Digestible Indispensable Amino Acid Score.

**Results:**

The protein MMD was 634 mg/kgBW/d, close to the current Estimated Average Requirement (660 mg/kgBW/d). Isoleucine and leucine MMD values were also close to current recommendations (21 and 40 mg/kgBW/d, respectively), whereas other MMDs were increased by 15%–150%. Oxidative losses were the largest contributor to the MMD (54%–72%) followed by endogenous gut losses, particularly for threonine (33%). The MMD for total IAAs as a proportion of protein (41%) was close to that of body protein (42%) and almost 50% greater than current recommendations (28%). MMD-based protein quality scores were reduced compared with those based on current recommendations.

**Conclusions:**

A factorial model for estimation of the MMD for protein and each IAA provides a mechanistic understanding of requirements and their sensitivity to metabolic processes. Each parameter is affected by variations in protein quantity and quality, and different physiological states. Additional data, when available, will enable model refinement.

## Introduction

Dietary protein and the indispensable amino acids (IAAs) that it provides are critical components to support health, playing essential roles in both the maintenance of body protein and metabolism [1]. The recommended dietary intake for each of protein and the IAAs has been established to provide for ongoing nitrogen (N) and IAA losses, respectively, thus enabling body N balance (NB) and the maintenance of body protein [2]. However, protein metabolism is inherently complex and incompletely understood, leading to criticism of these current protein and IAA requirement estimates for being too low [[3], [4], [5]]. There is an overall lack of consensus concerning dietary protein and IAA recommendations. Although the current recommendations represent the average minimum intake required for body protein maintenance, multiple factors influence protein metabolism within different population groups and at the individual level. In addition to age, lean body mass, physical activity level, and other factors, much emphasis has been placed in recent years on protein quality, that is, the ability of different protein sources to meet current requirements [[6], [7], [8]]. It is widely recognized that additional information is required to accurately determine and understand the required protein and IAA intakes, particularly across different populations and physiological states [[9], [10], [11]]. The development of a mathematical (factorial) model capable of both predicting the minimum demand for total protein and each amino acid (AA) by accounting for the complexities of underlying metabolic processes would make a valuable contribution to this understanding. Importantly, such a model also highlights the relative importance of the underlying metabolic processes, providing insight into current requirement values.

The factorial method has not been used for the estimation or understanding of dietary protein or IAA requirements in adult humans since the early recommendations [12,13]. These recommendations were based on the factorial summation of N losses from the urine, feces, integument (hair, skin, and nails), and other miscellaneous losses (such as menstrual fluid), when a protein-free diet is consumed. However, there is a difference between obligatory N losses measured in the protein-free state and the N intake required for balance when dietary protein is consumed [2]. This difference reflects metabolic adaptation to the protein-free state, inefficiencies in dietary protein digestion/absorption and metabolic utilization, and the importance of the rapidly turning over gastrointestinal system in AA metabolism. Thus, subsequent recommendations [2,14] have recognized that the estimation of protein and IAA requirements should relate to a protein-fed state. The current FAO/WHO recommendations [2] for dietary protein were derived from a meta-analysis [15] of empirical data from NB studies feeding low-, mixed-, and high-quality dietary protein sources. The current estimated average requirement (EAR) for dietary protein represents the minimum dietary intake of high-quality protein required for NB (0.66 g protein/kgBW/d) [2], and the “safe” requirement, which will ensure the minimum intake is met by 95% of the population, is 0.83 g of high-quality protein/kgBW/d. However, the NB method tends to underestimate N loss and overestimate N provision, and may underestimate N requirements because of an adaptation to low N intakes [2,4,16]. The current recommendations for the IAAs [2,17] are based, for the most part, on the use of stable isotopes and the indicator amino acid oxidation (IAAO) methodology, although for some IAAs, a reanalysis of early NB data has also been included. These methods have given rise to requirement estimates that are considerably higher than those obtained using the original NB data [14], but are also not without assumptions and limitations [2,[18], [19], [20]]. In particular, some approaches are based entirely upon oxidative losses, and do not take into account nonoxidative AA losses, for which some sources (such as the secretion of AAs into the gastrointestinal tract [21]) can be substantial [22]. Since these early experiments, however, much data from research aimed at both qualifying and quantifying sources of AA loss under protein alimentation, as well as characterizing the dynamics of human AA metabolism, have become available. Subsets of these data have been used in previously published quantitative models for the estimation of gut endogenous losses [21] and total oxidative losses (TOL) [23]. The integration of these models along with the estimation of each additional source of obligatory AA loss in humans consuming a protein-containing diet allows the calculation, using a factorial approach, of the minimum demand for dietary protein and each IAA at maintenance. In addition, as the available data include estimates for both IAAs and the dispensable amino acids (DAAs), total protein losses can be calculated as the sum of losses for total AAs, rather than derived from N losses.

**FIGURE 1:**
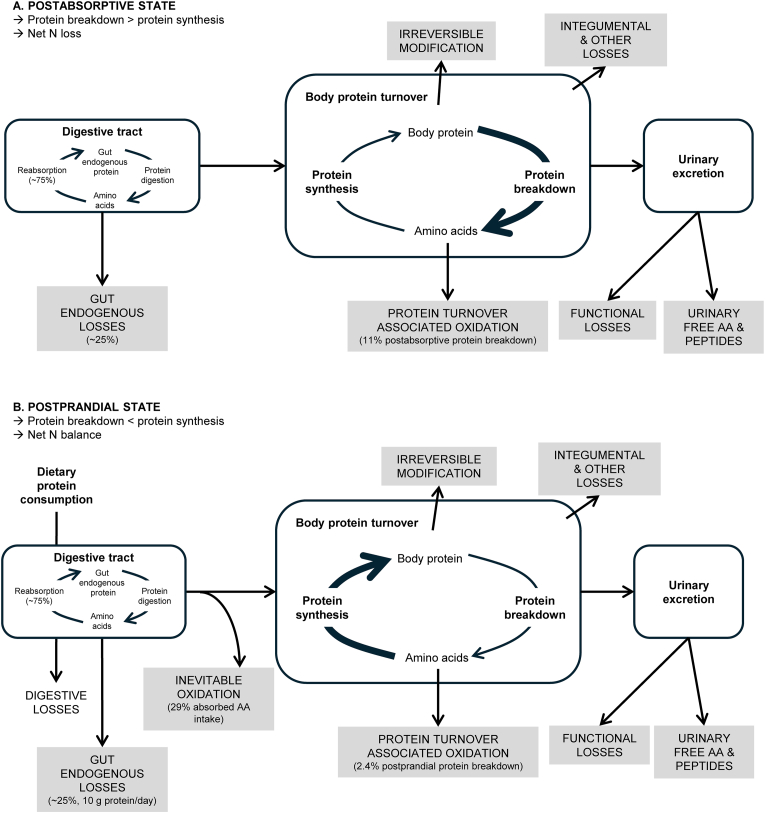
Obligatory losses of nitrogen (N) and amino acids (AAs) from the body that occur over the 24-h diurnal cycle (gray boxes), with specific losses characterizing each of the (A) postabsorptive (protein turnover–associated oxidation in the postabsorptive state) and (B) postprandial (protein turnover–associated oxidation in the postprandial state and inevitable oxidation) periods. Digestive losses are dependent on source of protein and are not considered obligatory.

Obligatory losses of N and AAs from the body occur over a diurnal cycle, with specific losses characterizing each of the postabsorptive (fasted) and postprandial (fed) states [24]. As detailed in Figure 1, these losses are centered around the process of protein turnover, the constant breakdown and resynthesis of body protein that ensures the supply of AAs for crucial tissues and organs, the recycling of AAs for new protein synthesis, and the maintenance of structural integrity of body proteins [1]. Although breakdown exceeds synthesis in the postabsorptive state, leading to a net negative NB, the dietary supply of AAs supports synthesis in excess of breakdown in the postprandial state, resulting in a net neutral balance over 24 h. In the postabsorptive state, the predominant source of AA loss is oxidation associated with protein turnover, with a previous model defining this as 11% of AAs released from protein breakdown (Figure 1A) [23], a value that may be influenced by the amount of protein intake in previous meals [25]. Oxidative losses associated with postabsorptive protein turnover occur primarily due to the provision of AAs to supply energy via gluconeogenesis, as well as inefficiency in the recycling of AAs from protein breakdown back to protein synthesis [26,27]. Recycling inefficiency is also present in the postprandial state, but apparently at a much-reduced rate (estimated at 2.4% of AAs released from protein breakdown [23]) because of the supply of dietary AAs (Figure 1B). The postprandial state is also characterized by inevitable oxidative losses because of the constitutive catabolism of each absorbed dietary AA at an estimated rate of ∼29% and relates to the first-pass gut tissue and liver AA catabolism [23]. The major nonoxidative route of loss is gut endogenous AA losses, because of the secretion of protein and AAs into the intestinal lumen. Although ∼75% of secreted AAs are reabsorbed after digestion, the daily loss of AAs via gut endogenous secretions has been modeled at 10.2 g protein/d and 70.1 mg IAAs/kgBW/d [21]. Gut endogenous losses are distinct from digestive losses because of inefficiency in the digestibility of dietary protein and subsequent absorption of AAs [21]. Additional nonoxidative losses include integumental (hair, skin, nails, and miscellaneous) losses, the urinary excretion of free AAs and peptides, functional losses via the synthesis of essential nonprotein compounds (such as the conversion of tryptophan into serotonin), and irreversible modification (such as hydroxylysine) [28,29]. Updated estimates for these additional losses, based on the collection and integration of data related to the consumption of a protein-containing diet, are required.

It is important to recognize that the requirements for dietary protein and each IAA, as laid out by the current recommendations, represent minimum intakes to support maintenance (that is, homeostasis) only, and do not account for the effects of dietary protein, the chemical composition of the diet, lean body mass, physical activity level, age, and other factors, on protein and AA metabolism. Furthermore, each protein source varies in the composition, digestibility, and availability of IAAs, a concept reflected in protein quality scores such as the Digestible Indispensable Amino Acid Score (DIAAS) [17]. The development and application of a factorial modeling approach enables the characterization of each source of loss individually, creating a model that is capable of addressing the multiple complexities of protein metabolism for application to different populations and physiological states in subsequent iterations. Such a model can also provide a framework to assess the relative importance of underlying metabolic processes as well as the mechanisms underpinning their variation.

This contribution describes the development of a mathematical (factorial) model for the prediction of the minimum metabolic demand (MMD) for IAAs and protein. The model is based upon the summation of maintenance IAA and protein losses from the human body, determined under conditions whereby subjects receive a protein-containing diet at amounts commensurate with current estimates of requirements. Previously published oxidative and gut endogenous losses are integrated with updated estimates for each additional source of loss: integumental (hair, skin, nails, and miscellaneous) losses; urinary free amino acids and peptides (UFAAP); functional losses; and irreversible modification; and all losses for each IAA and total protein are summed to calculate the MMD for protein and each IAA. MMD estimates have been compared with current recommended intakes. The primary aim in developing the model was to produce a baseline that can be further adapted to contribute to an understanding of the impact of different diets, populations, and physiological states on each source of loss. As a first investigation, the impact of dietary protein source on minimum demand estimates has been determined. The presented research highlights where a more complete understanding of IAA losses is needed and thus provides a blueprint for further research.

## Methods

### Conceptual summary

The objective of the factorial model is to predict the MMD for total protein and each IAA in the adult human male over a 24-h period. The intention of the model is to reflect a state of maintenance, where the intake of protein and each IAA is commensurate with requirements, and there is no imbalance between the supply compared with the need for total AAs, the IAA to DAA ratio, or each individual AA. Thus, the model describes a state where there is no oxidation of AAs because of supply in excess of requirements, nor because of protein synthesis being limited by AA imbalance. The current model also assumes that protein synthesis matches protein breakdown over 24 h so that there is no net loss nor gain of body protein, and that there is no additional demand for AAs to address states of ill-health or stress, such as immune responses, or repair and recovery. The model includes losses that occur after digestion and absorption of dietary protein, because of the influence of dietary protein type on AA availability. To meet these constraints and intention, data eligible for the model reflected the healthy adult male consuming an energy-replete diet consisting of high-quality protein consumed at or near the current EAR for protein. The overall objective is to provide basal estimates for the demand of total protein and each IAA that are able to be further adapted to provide an understanding of the impact of different diets, populations, and physiological states. The adult male was the initial focus of the modeling approach because of the majority of available data being on this population group across each source of loss, providing the most robust starting point compared with other demographics.

The factorial model describes the summation of each source of obligatory loss for protein and each IAA. For each source of obligatory loss, estimates were determined for each AA (including DAAs) and total protein loss was defined as the sum of individual AA losses. Obligatory losses occur over each 24-h period and are depicted in Figure 1 and described in Table 1. The sources of obligatory loss are as follows: TOL, given as the sum of individual estimates for protein-turnover–associated oxidation in the postabsorptive state (PTO_PA_), protein-turnover–associated oxidation in the postprandial state (PTO_PP_), and inevitable dietary AA oxidation (IO); gut endogenous losses (EGL); UFAAP; hair, skin, nails, and miscellaneous losses (HSNM); and, other losses (OL) because of irreversible modification and functional conversion. Oxidative losses depend on feeding state (postprandial or postabsorptive), a concept that has been covered in detail previously [23].

**TABLE 1:** Description of obligatory losses included in the factorial model, including losses specific to each of the postabsorptive and postprandial periods, as well as losses occurring throughout the 24-h period

Type of loss	Description	Collated estimate (previously published or undetermined)
Postabsorptive period only		
Oxidation associated with postabsorptive protein turnover	Due primarily to the provision of AAs to supply energy via gluconeogenesis, as well as inefficiency in recycling of AAs from protein breakdown back to protein synthesis [26,27].	11% of postabsorptive protein breakdown [23].
Postprandial period only		
Oxidation associated with postprandial protein turnover	Due primarily to inefficiency in recycling of AAs from protein breakdown back to protein synthesis [23].	2.4% of postprandial protein breakdown [23].
Inevitable oxidation	The catabolism of a proportion of each absorbed dietary AA because of constitutive enzymatic activity and first-pass catabolism [29,30].	29% dietary absorbed AAs [23].
24-h period		
Gut endogenous losses	Because of the secretion (and no reabsorption) of proteins, peptides, and AAs into the gastrointestinal lumen along the entire gastrointestinal tract [21].	10.2 g total protein/d [21].
Hair, skin, nails, and miscellaneous losses	Because of the shedding of hair, skin, nails, and other body tissues or fluids such as menstrual fluid [2,29].	Requires determination
Urinary free amino acids and peptides	Excretion of AAs in the urine either in free or bound form [29].	Requires determination
Other losses	Losses due to irreversible modification and nonprotein metabolic conversion [28,29].	Requires determination

Abbreviation: AA, amino acid.

Estimates of PTO_PA_, PTO_PP_, and IO (and therefore TOL) and EGL for each AA and total protein have been previously discussed [21,23] (Table 1). These data are predominantly specific to the adult human male, as previously described [21,23]. Although some female data were included in the estimation of EGL, the evidence to date suggests no difference between sexes. Full calculation of the MMD requires the determination of additional estimates for HSNM, UFAAP, and OL for each AA and total protein in the first instance, followed by summation of all losses to predict the MMD for that AA or total protein (as the sum of all AAs). The factorial model for the MMD for each AA and total protein was therefore developed in 2 steps:

Step 1: estimation of each additional source of obligatory loss for each AA and total protein.

A. HSNM
B. UFAAP
C. OL

Step 2: summation of obligatory losses (those estimated in Step 1 along with previously calculated losses) to determine the MMD for each AA and total protein.

### Formulas

The full factorial summation for the MMD of each AA is described by Equation *1*:

(1) MMD (AA) = TOL + EGL + UFAAP + HSNM + OL = (PTO_PA_ + PTO_PP_ + IO) + EGL + UFAAP + HSNM + OL

Equation *2* describes the summation of the MMD for each AA to determine the MMD for total protein.

(2) $$\text{MMD}\;\left(\text{total}\;\text{protein}\right)={\sum^{n}\;}_{i=1}\;{\left[({\text{PTO}}_{\text{PA}}\right)}_{i}+({{\text{PTO}}_{\text{PP}})}_{i}+{\left(\text{IO}\right)}_{i}+{\left(\text{EGL}\right)}_{i}+{\left(\text{UFAAP}\right)}_{i}+{\left(\text{HSNM}\right)}_{i}+{\left(\text{OL}\right)}_{i}]$$

where *i* is the *i*th AA and *n* refers to total AAs.

In Equation *3*, the previously published estimates for PTO_PA_, PTO_PP_, and IO have been incorporated into Equation *1*, where PB_PA_ and PB_PP_ are the rates of protein breakdown during the postabsorptive and postprandial periods, respectively. Published estimates for EGL are specific to each AA.

(3) MMD = (0.11PB_PA_ + 0.024PB_PP_ + 0.29MMR) + EGL + UFAAP + HSNM + OL

However, because IO represents the inevitable oxidation of each absorbed dietary AA, and the factorial model is based on a dietary intake of protein and IAAs that exactly supplies the metabolic requirement, IO is a proportion of MMD, necessitating the rearrangement of Equation *2* to solve for the MMD (Equation *3*):

MMD – 0.29MMD = 0.11PB_PA_ + 0.024PB_PP_ + EGL + UFAAP + HSNM + OL

(3) 0.71MMD = (0.11PB_PA_ + 0.024PB_PP_ + EGL + UFAAP + HSNM + OL) MMD = (0.11PB_PA_ + 0.024PB_PP_ + EGL + UFAAP + HSNM + OL)/0.71

The value of IO can then be determined as a proportion of MMD, according to

(4) IO = 0.29 × MMD

### Step 1: estimation of each additional source of obligatory loss for each AA and total protein

#### HSNM

Total HSNM N losses were set at 5 ± 0.9 mg N/kgBW/d as described in the 2007 FAO guidelines [2], and multiplied by 6.25 for conversion to total protein. These published values were based primarily on studies in young men and are negligibly affected when the data obtained in females and older men are omitted. To calculate losses for the individual essential amino acids (EAAs), the total HSNM value was separated into 2 components, dermal (skin, hair, and nail) losses and miscellaneous losses, as the AA composition of each of these components differ. According to the 2007 FAO guidelines [2], miscellaneous losses are 1.8 mg N/kgBW/d; the hair, skin, and nail component by difference was therefore taken to be 3.2 mg N/kgBW/d. Individual miscellaneous losses for the AAs were calculated using the AA composition of whole-body protein [17,31]. To calculate hair, skin, and nail losses for individual EAAs, mean data for the AA composition of human skin, hair, and nails [32] were used (Supplementary Table 1). Hair, skin, and nail losses were added to miscellaneous losses to give the total HSNM loss value for each AA. HSNM losses for the individual AAs were summed to determine the total protein HSNM loss value.

#### UFAAP

The values for UFAAP are presented as means ± SD, based on a number of studies where human data for the concentration of individual AAs present in the urine of healthy young male adults were provided [[33], [34], [35]] (Supplementary Table 2). In these early studies, AA excretion data for both free AAs, and those bound in peptides, were provided in units of mg/d, which were then divided by subject body weight to determine AA loss in units of mg/kgBW/d. More recent studies have provided urinary AA reference ranges as mmol/mol creatinine [[36], [37], [38], [39]], but these data refer to free AAs only. It was concluded that total urinary AA data (free and bound) were necessary for the model. However, early AA analyses were performed using microbiological assays, or early chromatographic methods, which may be less accurate than the advanced HPLC approaches used to generate more recent data. Values of free urinary AAs obtained from the early studies (bound values were omitted) were compared with more current reference ranges for urinary free AAs, and a high degree of correlation was found, with an *R*^2^ of 0.94 (data not shown). Therefore, the early datasets for free and bound AAs [[33], [34], [35]] were selected for use in the factorial model, with the mean and SD for each AA weighted for subject number in each study. As the early data did not present values for glutamine, urinary losses of glutamine were estimated from current reference ranges for urinary free AAs [37,39]. To convert current reference ranges (presented as mmol/mol creatinine) of glutamine to mmol, and subsequently mg/kgBW/d, a urinary excretion of 0.2 mmol/kgBW/d creatinine was used, the mean value of data presented in NB studies used to derive the current adult human protein requirement [2,15]. UFAAP losses for the individual AAs were summed to determine the total protein UFAAP loss value.

#### OL

OL include losses because of the irreversible modification of AAs after incorporation into proteins, rendering them nonusable for subsequent protein synthesis after breakdown, and losses because of the synthesis of essential nonprotein compounds, such as neurotransmitters. Although comparatively minor for most AAs, these OL contribute to the total IAA requirement and should not be overlooked. Previous reviews and models have described the major “OL” for the IAAs: irreversible modification of lysine to hydroxylysine, irreversible modification of histidine to 3-methyl histidine, incorporation of tryptophan into serotonin, and the role of methionine as a methyl donor in the body, essential for many reactions and processes [22,28,29]. In addition, tyrosine is the precursor to dopamine [40], increasing potential losses of and requirement for phenylalanine. Each source of “OL” was estimated independently, based on the urinary excretion of functional metabolites over 24 h, assuming a mol-to-mol ratio with the AA of focus. For example, it was assumed that the excretion of 1 mol of 5-hydroxyindoleacetic acid, the breakdown product of serotonin) represents the loss of 1 mol tryptophan. A description of all “OL” and the methods used for estimation are summarized in Table 2. In all cases, data were obtained from healthy adult participants consuming, or expected to be consuming (where specific details were not provided, such as the presentation of reference intervals), dietary protein in line with current recommended intakes. All data represent young males with the exception of studies where sex was not specified (irreversible lysine modification, pyrimidine metabolism), and for outcomes where sex-specific data were not available [functional roles of the sulfur amino acids (SAAs)]. For irreversible lysine modification and pyrimidine metabolism, there is no apparent difference between sexes in the excretion of hydroxylysine per kg of bodyweight [44] or β-aminoisobutyrate per unit of creatinine [75], respectively. For SAA roles, no 24-h data for nontaurine urinary sulfur metabolites specific to healthy young males consuming a protein or methionine intake close to current requirements were identified. Values from mixed populations of both healthy adults consuming an SAA-restricted diet were included for completeness [52,76]. Although an influence of sex on taurine excretion has been suggested [77], values for females are reduced in comparison with males, mitigating the possibility of overestimation of SAA losses because of this difference. OL values for the individual AAs were summed to determine the total protein OL value. Data used for the estimation of each source of OL are provided in Supplementary Tables 3–9.

**TABLE 2:** Summary of other losses (OL) of amino acids included in the factorial model, because of irreversible modification and the synthesis of essential nonprotein compounds, such as neurotransmitters

Amino acid	Sources of other loss	Methodology for estimation	References for estimation
IAA			
Histidine	Irreversible modification to 3-methyl histidine in the synthesis of contractile proteins [22].	Urinary excretion of 3-methylhistidine over 24 h.	[22,41,42]
Lysine	Irreversible modification to hydroxylysine in the synthesis of collagen proteins [29,43].	Urinary excretion of hydroxylysine (as free, glycosylated, and peptide-bound forms) over 24 h.	[44,[45], [46], [47], [48], [49], [50]]
SAA	Essential roles in: *1*) the methylation cycle as a methyl donor; and *2*) antioxidant production [29,51].	Urinary excretion of the major methionine and cysteine metabolites over 24 h: taurine, homocysteine, cystathionine, homolanthionine, lanthionine, and S-sulfocysteine. Does not include peptides such as glutathione, as these are accounted for within UFAAP losses.	[[52], [53], [54]]
AAA	Tyrosine is the precursor for the synthesis of catecholamines [40].	Urinary excretion of catecholamine metabolites over 24 h: norepinephrine, normetanephrine, VMA, dopamine, HVA, epinephrine, metanephrine.	[55]
Tryptophan	Precursor for the synthesis of serotonin and melatonin, and essential role in kynurenine metabolism, for the de novo synthesis of nicotinamide adenine dinucleotide (NAD+) [56].	Urinary excretion of serotonin and kynurenine metabolites over 24 h: 5-hydroxyindoleacetic acid, kynurenine, kynurenic acid, anthranilic acid, xanthurenic acid, 6-sulfatoxymelatonin.	[[57], [58], [59], [60], [61]]
DAA^1^			
Glycine	Losses because of creatinine and uric acid excretion, for which synthesis of creatine and purines, respectively, involve the glycine backbone [62,63]. Although the amidino group from arginine and a methyl group provided by methionine are also utilized for creatine synthesis, the backbone of these amino acids is retained in the body. Similarly, comparatively minor components of glutamine and aspartate are involved in purine biosynthesis.	Urinary excretion of creatinine over 24 h. Urinary excretion of uric acid over 24 h.	[[64], [65], [66], [67], [68], [69]] [[70], [71], [72]]
Aspartate	Losses because of pyrimidine metabolism. Aspartate is a precursor for pyrimidine synthesis [62].	Urinary excretion of β-isoaminobutyrate, an end product of pyrimidine metabolism [62]. β-Alanine, also an end product, is accounted for within UFAAP.	[73]

Abbreviations: AAA, aromatic amino acid (phenylalanine and tyrosine); DAA, dispensable amino acid; HVA, homovanillic acid; SAA, sulfur amino acid (methionine and cysteine); UFAAP, urinary free amino acid and peptide; VMA, vanillylmandelic acid.

1 Numerous roles, many of which involve reversible/cyclic modification. Focus has been placed on predominant urinary losses. Loss of DNA and RNA in the urine is negligible compared with the other losses presented here [74].

### Step 2: summation of obligatory losses to determine the MMD for each AA and total protein

For each AA and total protein, the MMD was calculated according to Equation *3*, and a numerical value for IO was determined according to Equation *4*. The MMD values for total protein and each IAA were compared with current FAO/WHO recommendations [2,17]. Protein quality scoring patterns were calculated as mg/g total protein, by dividing the requirement or demand estimate for each IAA (mg/kgBW/d) by the total protein requirement (as g/d). In addition, the AA composition of the MMD (as mg/g total protein) was compared with the AA composition of total body protein.

### Sensitivity analysis

The effect of variation in each parameter of the factorial model (IO, PTO_PA_, PTO_PP_, EGL, UFAAP, HSNM, and OL) on the MMD for total protein, each IAA, and total DAAs was determined by altering the magnitude of each loss parameter independently and in 5% increments, up to a total variation range of *±*20%. The MMD was then plotted as a function of variation for each parameter and the slope of the line determined. The slope of the line (*b*) represents the amount of change in the MMD per each 1% variation in an obligatory loss parameter.

### Influence of dietary protein source on minimum requirements

The MMD for total protein is the predicted MMD associated with the provision of each IAA at required amounts after digestion and absorption, in a diet where both total protein and each AA are consumed at amounts commensurate with requirements. In contrast, dietary protein sources vary by both AA composition and digestibility, impacting the amount of dietary protein that needs to be consumed to satisfy the metabolic demand (MMD) for each IAA. This has been described many times previously by the concept of protein quality, most recently the DIAAS, which compares the amount of each absorbed IAA provided by a protein source to a reference level, as per current IAA requirements [17]. To understand the ability of different dietary protein sources to provide the MMD for each IAA, the MMD values for each IAA and total protein (as predicted by the factorial model) were used as reference patterns for the DIAAS to calculate MMD-based protein quality scores (DIAAS-MMD). The selection of protein sources was based on the availability of both true ileal digestibility (TID) and AA composition data, as well as ensuring variation across protein types. To account for variation in the literature, AA composition was determined as the mean of multiple datasets: whole milk [[78], [79], [80], [81]], whole egg [78,82], beef [83,84], soy protein isolate [78,85,86], rice [78,85], and mung bean [87,88]. Where values for tryptophan were not provided, tryptophan composition data from the USDA food composition database were used. Published TID values were sourced [89], with mean values used where multiple datasets were presented. TID values for individual AAs were used where available, otherwise the TID for all available AAs (mean), total protein, or total N served as a proxy. AA composition and TID data used for calculation of DIAAS scores are provided in Supplementary Table 10. DIAAS-MMD scores were compared with traditional DIAAS scores calculated using current IAA and protein requirements and the adult scoring pattern [17].

### Statistical analysis

All data plotting, trendline fitting, and statistical analyses were carried out using Microsoft Excel (version 2407) or SPSS (version 29.0.2.0) software. Where applicable, parameter estimates and model predictions are shown as mean values *±* SD. Where data were summed to calculate total values, the overall SD was determined as the square root (SQRT) of the sum of the squares of all parameter SDs. For example, the SD of each MMD was calculated as follows:

SD(MMD) = SQRT[SD(IO)^2^ + SD(PTO_PA_)^2^ + SD(PTO_PP_)^2^ + SD(EGL)^2^ + SD(UFAAP)^2^ + SD(HSNM)^2^ + SD(OL)^2^]

## Results

### Factorial predictions of minimum metabolic requirements for total protein and each AA

**FIGURE 2:**
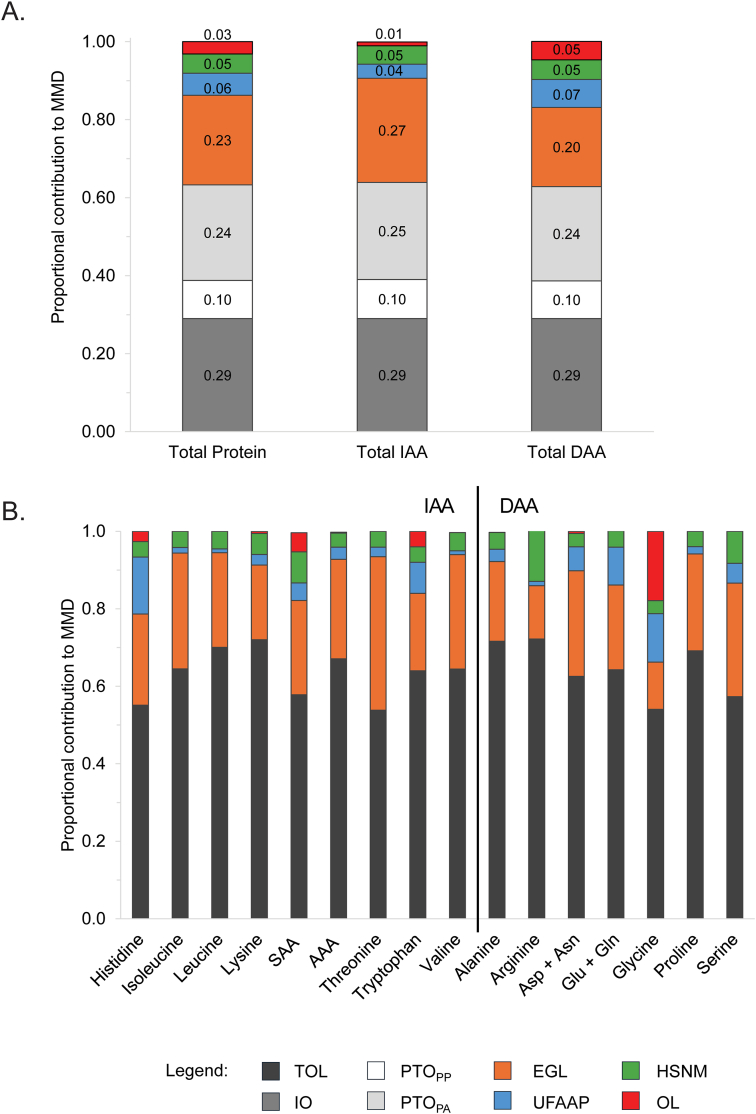
Proportional contribution of each source of obligatory loss to the MMD for (A) total protein, total IAAs, and total DAAs; and (B) each AA. In (B), total oxidative losses (TOL) represent the sum of IO, PTO_PP_, and PTO_PA_. AA, amino acid; AAA, aromatic amino acid; Asn, asparagine; Asp, aspartic acid; DAA, dispensable amino acid; EGL, gut endogenous loss; Glu, glutamic acid; Gln, glutamine; HSNM, hair, skin, nails, and miscellaneous loss; IAA, indispensable amino acid; IO, inevitable oxidation; MMD, minimum metabolic demand; OL, other loss; PTO_PA_, oxidation associated with postabsorptive protein turnover; PTO_PP_, oxidation associated with postprandial protein turnover; SAA, sulfur amino acid; UFAAP, urinary free amino acid and peptide.

MMD values predicted by the factorial model for total protein and each AA are presented in Table 3. The MMD for total protein was 634.1 ± 62.6 mg/kgBW/d, comprised total IAA and DAA MMD values of 262.3 ± 26.5 and 371.8 ± 36.2 mg/kgBW/d, respectively. The MMD for individual IAAs ranged from 7.5 ± 0.7 (tryptophan) to 41.6 ± 4.7 [aromatic amino acid (AAA)] mg/kgBW/d. The DAA with highest MMD was glycine, at 96.0 ± 8.0 mg/kgBW/d. For total protein and each of the IAAs and DAAs, the greatest source of loss (as a proportion of MMD) was 24-h oxidative losses [sum of IO (29%), PTO_PP_ (10%), and PTO_PA_ (24%–25%)], followed by EGL (20%–27%), with minor contributions from UFAAP (4%–7%), HSNM (5%), and OL (1%–5%) (Figure 2A). However, there was variation in loss contributions between the AAs (Figure 2B). Among the major sources of obligatory loss, oxidative losses showed the highest contribution to the MMD for lysine, alanine, and arginine (72%), followed by leucine (70%) and proline (69%), whereas the contribution of EGL to the threonine MMD was ≥33% higher in comparison with other AAs (40% compared with 12%–30%). Minor sources of loss (as a proportion of the MMD) were highest for histidine (UFAAP, 15%), SAA (HSNM, 8% and OL, 5%), arginine (HSNM, 13%), glycine (UFAAP, 13% and OL, 18%), and serine (HSNM, 8%).

**TABLE 3:** Factorial model^1^ for the prediction of the MMD for protein and AAs in the adult human male

Protein/AA	Oxidative losses^2^			Nonoxidative losses				MMD^8^
	IO^3^	PTO_PP_	PTO_PA_	EGL^4^	UFAAP^5^	HSNM^6^	OL^7^	
	mg/kgBW/d							
Total protein^9^	183.9 (12.7)	62.2 (51.8)	155.1 (28.2)	145.6 (19.6)	35.9 (3.7)	31.3 (5.6)	20.1 (2.7)	634.1 (62.6)
IAA								
Histidine	6.5 (0.5)	1.7 (1.4)	4.2 (0.8)	5.3 (1.2)	3.3 (0.67)	0.9 (0.2)	0.6 (0.2)	22.5 (2.1)
Isoleucine	6.2 (0.4)	2.2 (1.8)	5.4 (1.0)	6.4 (0.9)	0.3 (0.02)	0.9 (0.2)	—	21.4 (2.3)
Leucine	11.5 (0.8)	4.7 (3.9)	11.6 (2.1)	9.7 (1.3)	0.4 (0.05)	1.8 (0.3)	—	39.7 (4.6)
Lysine	10.7 (0.7)	4.5 (3.8)	11.3 (2.1)	7.1 (0.8)	1.0 (0.2)	2.0 (0.4)	0.2 (0.04)	36.8 (4.4)
SAA	7.6 (0.5)	2.2 (1.8)	5.4 (1.0)	6.4 (0.4)	1.2 (0.3)	2.1 (0.4)	1.3 (1.0)	26.3 (2.4)
AAA	12.1 (0.8)	4.5 (3.8)	11.3 (2.1)	10.7 (1.8)	1.3 (0.2)	1.5 (0.3)	0.1 (0.03)	41.6 (4.7)
Threonine	10.6 (0.7)	2.6 (2.2)	6.5 (1.2)	14.5 (1.9)	0.9 (0.1)	1.5 (0.3)	—	36.6 (3.1)
Tryptophan	2.2 (0.2)	0.7 (0.6)	1.9 (0.3)	1.5 (0.1)	0.6 (0.1)	0.3 (0.1)	0.3 (0.05)	7.5 (0.7)
Valine	8.6 (0.6)	3.0 (2.5)	7.6 (1.4)	8.8 (0.8)	0.3 (0.1)	1.4 (0.2)	—	29.8 (3.0)
Total IAA	76.1 (5.2)	26.2 (21.8)	65.3 (11.9)	70.1 (9.0)	9.4 (0.8)	12.4 (2.2)	2.6 (1.0)	262.3 (26.5)
DAA								
Alanine	10.0 (0.7)	4.2 (3.5)	10.5 (1.9)	7.1 (0.7)	1.1 (0.4)	1.5 (0.3)	—	34.5 (4.1)
Arginine	10.5 (0.7)	4.5 (3.7)	11.2 (2.0)	5.0 (0.3)	0.4 (0.1)	4.8 (0.9)	—	36.3 (4.3)
Asp + Asn	15.9 (1.1)	5.3 (4.4)	13.2 (2.4)	15.0 (1.1)	3.4 (0.3)	1.9 (0.3)	0.3 (0.1)	55.0 (5.2)
Glu + Gln	22.0 (1.5)	7.6 (6.4)	19.1 (3.5)	16.6 (3.2)	7.4 (1.4)	3.2 (0.6)	—	75.8 (8.1)
Glycine	27.8 (1.9)	6.9 (5.7)	17.2 (3.1)	11.7 (3.0)	12.0 (2.7)	3.2 (0.6)	17.2 (2.3)	96.0 (8.0)
Proline	12.4 (0.9)	4.9 (4.1)	12.3 (2.2)	10.7 (0.9)	0.8 (0.3)	1.7 (0.3)	—	42.8 (4.8)
Serine	9.1 (0.6)	2.5 (2.1)	6.4 (1.2)	9.2 (1.3)	1.6 (0.5)	2.6 (0.5)	—	31.4 (2.8)
Total DAA	107.8 (7.4)	36.0 (30.0)	89.8 (16.3)	75.5 (10.7)	26.6 (3.6)	18.8 (3.4)	17.5 (2.3)	371.8 (36.2)

Abbreviations: AA, amino acid; AAA, aromatic amino acid; Asp, aspartic acid; Asn, asparagine; DAA, dispensable amino acid; Glu, glutamic acid; Gln, glutamine; IAA, indispensable amino acid; SAA, sulfur amino acid.

1 The model is defined by Equations *1–4* as described in Methods.

2 Oxidative losses, including losses associated with inevitable oxidation (IO), postprandial protein turnover (PTO_PP_), and postabsorptive protein turnover (PTO_PA_), have been previously determined and described in full [23].

3 IO was defined as 29% of absorbed dietary AAs (represented by the MMD) and was calculated after determination of the value of MMD for total protein and each AA.

4 Endogenous gut losses (EGL) have been previously determined and described in full [21].

5 Urinary free AAs and peptides (UFAAP) were an average of published data [[33], [34], [35]], divided by 75 kg to convert to mg.kg/BW/d where necessary.

6 Hair, skin, nails, and miscellaneous (HSNM) losses were calculated based on the value of 5 ± 0.9 mg N.kgBW/d as described in the 2007 FAO guidelines [2], comprised are 1.8 mg N.kgBW/d miscellaneous losses and 3.2 mg N N.kgBW/d hair, skin, and nail losses. Miscellaneous and hair, skin, and nail losses for each AA were calculated using the AA composition of whole-body protein [17,31], and mean data for the AA composition of human skin, hair, and nails [32], respectively (Supplementary Table 1).

7 Other losses (OL) were defined as losses because of the irreversible modification of AAs after incorporation into proteins and the synthesis of essential nonprotein compounds. Data used for the estimation of each source of other AA loss are provided in Supplementary Tables 3–7.

8 The minimum metabolic demand (MMD) for total protein and each AA was calculated as the sum of all individual losses with the exception of IO, divided by 0.71, as described by Equation *3*.

9 Values for total protein are the sum of values for all AAs, with the exception of IO, which was calculated as a 29% of the MMD for protein, and HSNM, for which the value of 5 mg N.kgBW/d was used.

### Comparison of MMD predictions with current requirements and total body protein

Factorial model predictions for the minimum metabolic (absorbed) demand for total dietary protein and each IAA are compared with current estimates of requirements [2,17] in Table 4. Scoring patterns for each set of requirements (as mg/g protein) have also been determined and compared with the composition of body protein. Although the MMD for total protein was similar to the current EAR for protein (634 compared with 660 mg/kgBW/d), total IAA metabolic requirements predicted by the factorial model (262 mg/kgBW/d) were ∼40% greater than current IAA requirements (184 mg/kgBW/d). MMD values for some individual IAAs were comparable with current IAA requirements, specifically the branched chain AA isoleucine (21 compared with 20 mg/kgBW/d), and leucine (40 compared with 39 mg/kgBW/d), whereas valine (30 compared with 26 mg/kgBW/d) and lysine (37 compared with 30 mg/kgBW/d) show 15% and 23% increases, respectively, compared with current recommended intakes. The model predicted MMD for the remaining IAAs is notably higher than current recommendations, however, with an increase ranging from 70% (SAA and AAA) to 250% (threonine). These differences are reflected in the scoring patterns calculated for each set of requirements, as well as the notable difference in the magnitude of total IAA as a proportion of the total protein requirement, at 41% of the predicted MMD for protein, compared with 28% for the sum of current IAA requirements as percentage of the EAR for total protein. The proportion of 42% total protein predicted by the factorial model closely reflects the IAA proportion of total body protein (42%), with additional congruency in the proportion of some individual IAAs, primarily isoleucine (33 compared with 35 mg/g protein), tryptophan (13 compared with 12 mg/g protein), and valine (47 compared with 49 mg/g protein). For all other IAAs, the scoring pattern predicted by the factorial model is closer to the composition of body protein than that of current requirement estimates.

**TABLE 4:** Comparison of factorial model predictions for the MMD of total dietary protein and each IAA to current requirements and the composition of body protein

Protein/AA	Demand/requirement^1^		Scoring pattern^2^		Body protein^3^
	Factorial model	FAO/WHO 2007	Factorial model	FAO/WHO 2007	
	mg/kgBW/d		mg/g protein		
Total protein	634	660	1000	1000	1000
Histidine	23	10	36	15	27
Isoleucine	21	20	33	30	35
Leucine	40	39	63	59	75
Lysine	37	30	58	45	73
SAA	26	15	41	23	35
AAA	42	25	66	38	73
Threonine	37	15	58	23	42
Tryptophan	8	4	13	6	12
Valine	30	26	47	39	49
Total IAA	262	184	413	279	421
Total DAA	372	476	587	721	579
IAA:DAA	—	—	41:59	28:72	42:58

Abbreviations: AAA, aromatic amino acid; DAA, dispensable amino acid; EAR, estimated average requirement; IAA, indispensable amino acid; SAA, sulfur amino acid.

1 Demand and requirements represent the MMD for total protein and each IAA as predicted by the factorial model, and current requirements for total protein (EAR) and each IAA as outlined in the most recent FAO/WHO reports [2,17], respectively.

2 Scoring patterns were calculated by dividing the requirement for each IAA by the requirement for total protein.

3 AA composition of whole-body protein [17,31].

### Sensitivity analysis

The impact of change in any one parameter of the factorial model on MMD for total protein, total IAAs, total DAAs, and each IAA is provided in Supplementary Table 11. Regardless of the category, a change in TOL (the sum of IO, PTO_PP_, and PTO_PA_) had the largest impact on MMD. Within TOL, changes in IO made the largest impact on the MMD on total protein, total IAAs, total DAAs, and the majority of individual AAs, followed by changes in PTO_PA_. Exceptions were leucine, lysine, alanine, and arginine. Changes in EGL had a notable impact on the MMD, in some cases comparable with that because of changes in IO and/or PTO_PA_. In particular, changes in EGL created the largest impact on the MMD for threonine (0.145 mg/kgBW/d per each 1% change in EGL) of any parameter, with a similar finding for isoleucine and valine (0.064 and 0.088 mg/kgBW/d per each 1% change in EGL, respectively). Changes in PTOPP, UFAAP, and HSNM had lower impacts on the MMD across all categories, with changes in OL having the smallest effect of all parameters.

### Effect of dietary protein source on minimum protein and IAA requirements

**FIGURE 3:**
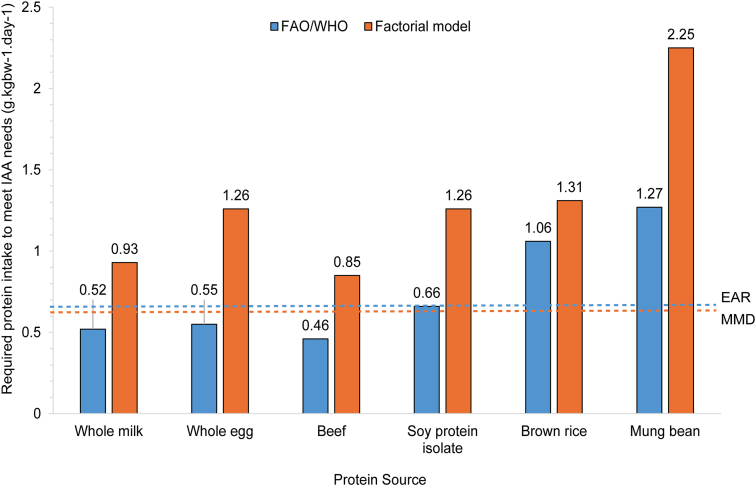
Calculation of protein intake required to meet IAA needs according to current requirements [2,17], (FAO/WHO, blue bars) and requirements predicted by the factorial model (MMD, orange bars) based on protein quality score (DIAAS or DIAAS modified to use the MMD values predicted by the factorial model, respectively). The total protein demand or requirement (EAR or MMD, respectively) is shown as a dashed horizontal line. DIAAS, Digestible Indispensable Amino Acid Score; EAR, estimated average requirement; MMD, minimum metabolic demand.

In Table 5, MMD-adjusted protein quality scores (DIAAS-MMD) have been calculated and compared with the traditional DIAAS (based on current adult requirements) for a range of dietary protein sources. Regardless of dietary protein source, protein quality, reflected in the lowest obtained reference ratio, was lower when the MMD scoring pattern was used to calculate the DIAAS. These decreases in protein quality score compared with calculations using current protein and IAA requirement estimates ranged from 23% (rice) to 58% (whole egg). Although all plant protein sources had a DIAAS <1 for both scoring patterns, the DIAAS of animal protein sources also dropped to below 1 when the MMD scoring pattern was used. Calculation of protein needs based on protein quality score (EAR or MMD divided by DIAAS or DIAAS-MMD, respectively) suggested that, according to the factorial model, both animal and plant protein sources would need to be consumed at increased amountss (relative to current requirements) to sufficiently meet the metabolic demand for all IAAs (Figure 3). For example, although whole milk protein may sufficiently provide for the IAA needs of an adult at an intake of 0.52 g/kgBW/d according to current protein and IAA requirements, an intake of 0.93 g/kgBW/d is predicted to be necessary according to the factorial model. Similarly, if consumed as the sole protein source, protein quality scores calculated using the MMD scoring pattern indicate that 0.85 g/kgBW/d beef protein (compared with 0.46 g/kgBW/d) and 1.26 g/kgBW/d whole egg protein (compared with 0.55 g/kgBW/d) would be required. For plant protein sources, MMD-associated intakes ranged from 1.26 g/kgBW/d for soy protein isolate, to 1.31 g/kgBW/d for rice protein, and 2.25 g/kgBW/d for mung bean protein. Although the first-limiting AA (that with the lowest reference ratio) was the same for plant protein sources across both scoring patterns (MMD and FAO/WHO), there were changes for animal protein sources. The first-limiting AA shifted from SAA (FAO/WHO) to threonine (MMD) for whole milk, and from leucine to threonine for beef. For egg, both valine and histidine were found to be first-limiting against the FAO/WHO scoring pattern, whereas only histidine was first-limiting against the MMD scoring pattern.

**TABLE 5:** Protein quality scores for a range of protein sources calculated based on DIAAS using the scoring patterns for each of the factorial model (MMD) and current requirements (FAO)

IAA	Scoring pattern^1^		Reference ratio^2^											
	MMD	FAO	Whole milk		Egg		Beef		Soy protein isolate		Rice		Mung bean	
	mg/g intake		MMD	FAO	MMD	FAO	MMD	FAO	MMD	FAO	MMD	FAO	MMD	FAO
Histidine	36	15	0.77	1.84	0.50	1.19	1.02	2.44	0.63	1.60	0.54	1.30	0.68	1.62
Isoleucine	33	30	1.2	1.32	1.25	1.38	1.56	1.72	1.07	1.27	1.03	1.13	1.01	1.11
Leucine	63	59	1.4	1.5	1.14	1.21	1.33	1.42	1.10	1.25	1.13	1.21	1.00	1.07
Lysine	58	45	1.34	1.72	1.06	1.37	1.52	1.96	0.97	1.24	0.48	0.62	0.90	1.17
SAA	41	22	0.69	1.28	0.88	1.64	1.04	1.94	0.50	1.00	0.88	1.64	0.28	0.52
AAA	66	38	1.4	2.43	1.17	2.03	1.16	2.01	1.22	2.24	1.39	2.42	1.12	1.95
Threonine	58	23	0.68	1.7	0.66	1.66	0.74	1.87	0.56	1.52	0.55	1.39	0.40	1.00
Tryptophan	13	6	0.78	1.69	0.74	1.61	0.92	1.98	0.88	2.00	0.94	2.04	0.55	1.20
Valine	47	39	1.06	1.28	0.99	1.19	1.25	1.51	0.79	1.03	0.98	1.18	0.83	1.01
Lowest reference ratio, DIAAS			0.68	1.28	0.50	1.19	0.74	1.42	0.50	1.00	0.48	0.62	0.28	0.52

Abbreviations: AAA, aromatic amino acid; DIAAS, Digestible Indispensable Amino Acid Score; MMD, minimum metabolic demand; SAA, sulfur amino acid; TID, true ileal digestibility.

1 Scoring patterns were calculated by dividing the demand or requirement for each IAA by the demand or requirement for total protein. Demand and requirement values are provided in Table 4.

2 The reference ratio for each IAA for each protein source was calculated by expressing the digestible IAA composition of each protein source [90], as a proportion of the scoring pattern for each of the factorial model (MMD) and FAO/WHO (FAO) requirements [2,17]. Data showing the IAA composition and TID for each protein source are provided in Supplementary Table 9.

## Discussion

For the first time in over 50 y, a mathematical (factorial) model for the estimation of the minimum demand for protein and IAAs has been developed and is presented here. The model predicts the MMD after digestion and absorption for total protein and each IAA using published data from research aimed at both qualifying and quantifying sources of AA loss under protein alimentation, as well as characterizing the dynamics of human AA metabolism. Although the predicted MMD for protein is similar to the current requirement estimate (0.634 compared with 0.660 g/kgBW/d, respectively), model predicted values for the IAAs are notably higher than current recommendations overall, highlighting key differences in the estimated ratio required between IAAs and DAAs to support the maintenance of body protein. These differences translate into notably higher protein intake requirement estimates for all protein sources when applied to DIAAS scoring patterns, in line with the consensus that minimum intakes for protein are higher than the current EAR [[3], [4], [5]]. The factorial model not only provides insights into the MMD for protein, but also the AA composition of that demand, and the relative contribution of the different processes describing protein and AA metabolism. The factorial model is novel in its applicability to a protein-containing diet, as well as the flexibility to adapt to different diets, populations, and physiological states, which will be realized in further iterations. Furthermore, the model provides insight into minimum estimates for the metabolic demand of each DAA, which to our knowledge, has never been carried out for humans. The model fills a critical gap in the literature [9] and makes a valuable contribution to the current understanding of protein and IAA requirements.

The factorial model aims to provide a basal estimate of the demand for protein and each IAA after the digestion and absorption of a protein source and is based on a hypothetical situation where digestible intake exactly meets requirements for protein and each individual AA. It is well established, however, that the source and magnitude of dietary protein consumed has a significant impact on subsequent protein and IAA metabolism, and thus the estimated requirement for protein and each IAA, creating difficulty in achieving an accurate understanding of both the true demand and minimum requirements. For example, IO, which relates to the first-pass obligatory gut tissue and liver AA catabolism, occurs at an estimated rate of 29% of each absorbed dietary AA [23]. An increase in dietary protein intake will therefore increase the amount of IO and subsequently, the required dietary intake. Conversely, IO may also decrease with decreasing intake of AAs [30], suggesting adaptation to low protein states to account for AA inadequacy. Thus, any model aiming to isolate metabolic needs must perform independently of protein intake. At the same time, given the intimate relationship between dietary protein and IAA intake and metabolism, and the differences in metabolic processes between the fed and fasted states (diurnal metabolism), the same model must still consider and account for protein consumption. Many parameters of the presented factorial model have been based on data obtained under the conditions of protein alimentation in or around the currently accepted minimum requirements for protein and/or IAAs, which introduces the possibility that the magnitude of the included obligatory losses may bias the model toward these intakes. However, the relationship between protein intake and total obligatory losses is nonlinear, with the most meaningful differences observed between very low intakes and intakes reflecting NB and/or the breakpoint of IAAO methodology. In addition, for some sources of obligatory loss, including oxidation associated with protein turnover in the fasted state (PTO_PA_), the magnitude of loss remains consistent over a large intake range. Thus, the model has been constructed to describe obligatory metabolic losses per each AA within a physiological state that best represents the conditions of general protein feeding at a level that approximates the requirement, a state that has been shown to have a negligible impact on any one source of obligatory loss. The MMD estimated by the factorial model, therefore, has little reliance upon, and is minimally affected by, any intake other than the intake that meets metabolic requirements yet can also respond to changes in dietary protein and IAA intake. The model can therefore predict both minimum metabolic needs and intake requirements across different intakes of protein quality and quantity, as needed. Subsequent iterations and presentations of the model will highlight how differences in age, sex, activity level, muscle mass, dietary characteristics and more, interact to further affect minimum metabolic and intake requirements.

The primary finding in comparison of factorial predictions with current requirement estimates was a notable difference in minimum IAA needs, as well as the ratio between IAAs and DAAs, despite a similar minimum demand for total protein. For example, application of a TID for high-quality protein of 95% to the MMD produces a required dietary intake equivalent to the current EAR. However, total IAAs made up 41% of the protein MMD, compared with 28% for current recommendations [2,17]. This ratio (41:59) is closer to both the IAA:DAA ratio of human body protein (42:58) [17,31], and published ideal dietary ratios for simple-stomached animals (mean value of 55%–60% IAAs) [8,91,92], than that derived from current recommended values. If the predictions made by the factorial model are correct, protein quality scores calculated using current requirement estimates for the IAAs and total protein are too high and overestimate the ability of even “high” quality protein sources to provide adequate levels of the IAAs. When protein quality scores (DIAAS) were calculated using model predicted MMD values and subsequently translated into a minimum dietary intake requirement for each protein source, a minimum intake of 0.85 g/kgBW/d was required (beef), almost double that calculated using current requirement estimates for the IAAs (0.46 g/kgBW/d). However, the predicted (modeled) intake requirement for high-quality protein sources, ranging from 0.85 to 1.26 g/kgBW/d, was consistent with both the current recommended daily allowance (RDA) for protein (0.83 g/kgBW/d) and a reassessment of NB data using 2-phase linear regression (EAR and RDA of 0.91 and 0.99 g/kgBW/d, respectively) [4], and estimates using IAAO (EAR and RDA of 0.93 and 1.2 g/kgBW/d, respectively) [4]. A similar pattern was observed for lower quality protein sources, with predicted dietary intake requirements over 2 g/kgBW/d for some proteins (mung bean). These findings have important implications for protein and IAA requirements and dietary recommendations globally and warrant further investigation in experimental settings. It is important to highlight that the normal human diet contains a variety of different protein sources and foods, whereas protein quality scores via DIAAS reflect single protein sources. In addition, there is limited understanding of the effect of nonprotein nutritional components within the diet on protein and IAA requirements and protein quality [6]. For example, in addition to an influence of the amount dietary protein intake on endogenous gut losses, the presence and amount of dietary fiber and antinutritional factors within the diet increase endogenous gut losses and can decrease protein and IAA digestibility [93,94]. Conversely, proteases present in fruits may increase the provision of IAAs to the body [95]. Furthermore, carbohydrate and fat have been shown to influence the protein synthetic response to a meal [6]. By accounting for the diurnal nature of feeding, as well as the influence of dietary factors on processes underpinning protein and IAA metabolism, the factorial model lends itself to further investigations that can contribute to an understanding of protein quality and protein and IAA requirements in the context of the total diet, and at the level of the individual.

Within the overall greater demand for the IAAs predicted by the factorial model, there was a difference in their relative composition relative to current recommendations. Although the metabolic demand for some IAAs was in line with current requirement estimates, others showed over 2-fold increases, the nature of which can readily be investigated by examining the different components of the model making up each MMD. For example, MMD values for isoleucine (21 compared with 20 mg/kgBW/d), and leucine (40 compared with 39 mg/kgBW/d), are notably comparable with current requirement estimates. For these IAAs, the primary source of loss was oxidative losses followed by endogenous gut losses, with very small or no contributions from UFAAP, HSNM, and OL. A similar pattern can be observed for valine (30 compared with 26 mg/kgBW/d) and lysine (37 compared with 30 mg/kgBW/d), which had predicted demands 15% and 23% greater than current recommendations, respectively. In contrast, the MMD for threonine was 2.5-fold greater than the current requirement estimate, primarily due to substantial losses across the gastrointestinal tract (EGL, 14.5 mg/kgBW/d), almost equivalent to the current recommended intake for threonine (15 mg/kgBW/d) alone. This additional loss can be explained by the fact that threonine comprises a substantial proportion of mucin proteins forming the protective mucous layer of the gastrointestinal tract that is subject to a high degree of turnover [21]. However, TOL for threonine (19.7 mg/kgBW/d) were also independently greater than the current requirement estimate by 30%. In DIAAS calculations, threonine became the first-limiting AA for both whole milk (rather than SAA) and beef (rather than leucine) using MMD compared with FAO AA scoring patterns, respectively. Together, these data indicate that further examination regarding the role of and need for threonine in human health and dietary recommendations is required. Other IAAs showing much higher MMD values in comparison with current recommendations were histidine (2.3-fold increase) and tryptophan (almost 2-fold increase), also explained by losses over and above those because of oxidation. Histidine was found to have a high rate of excretion in the urine as either histidine (UFAAP, 3.3 mg/kgBW/d) or methylated derivatives such as 3-methyl histidine (OL, 0.6 mg/kgBW/d), making up almost 20% of the histidine MMD. Urinary histidine excretion has been shown to be high even in a protein-free state [33] and may be dependent on previous intake [96]. In addition, pregnant women excrete more histidine in their urine than nonpregnant women, possibly due to a higher turnover of hemoglobin [97], suggesting that histidine needs are sensitive to both diet and physiological state. For tryptophan, nonoxidative losses made up 36% of the MMD. Although oxidative losses are the major source of loss for the IAAs, the contribution ranges from 54% to 72% of the MMD, indicating that nonoxidative losses can account for ≤46% of the need for an IAA. Consistent with early discussions regarding the limitations of requirement estimates based entirely on oxidation measures [22], the factorial model highlights the importance of understanding and accounting for nonoxidative sources of loss, primarily (but not limited to) endogenous gut losses, which can have a significant impact on metabolic demand and subsequent requirement estimates. It is worth noting that the MMD for leucine has not considered the intake of leucine required to maximize protein synthesis, a modification that can be included in further iterations of the model, including differences in this requirement across population groups.

A novel feature of the present factorial model is the estimate of minimum obligatory losses for each of the DAAs. Traditionally, consideration for the DAAs has been omitted from dietary recommendations because of their endogenous production and nonlimiting nature regarding protein synthesis. Although animal science literature indicates that feed containing as low as 33% DAAs supports maximum growth and muscle development [92], the factorial model identified the loss of DAAs at 59% of total obligatory metabolic losses (the MMD for protein). Regardless, given that dietary protein ranges from 50% to 70% DAAs, and most excess dietary IAAs can be transaminated to provide DAAs, it is unlikely that dietary DAAs will be limiting for protein synthesis. However, importance has been placed on the DAAs for their role in functional processes outside of protein synthesis [51] and some DAAs have been described as “conditionally essential,” showing the ability to become limiting in certain conditions, such as arginine, proline, and glycine for preterm human infants and weanling neonates [51]. Dietary glycine has also been suggested to be rate-limiting for glutathione synthesis, particularly in those consuming low protein diets or during aging [98], and is fundamental for both creatine and purine synthesis [62,63]. It is worth noting that the factorial model predicted glycine to have the largest MMD of all the AAs (96 mg/kgBW/d) with 31% of the MMD because of OL (as urinary creatinine and uric acid) and urinary free glycine or glycine peptides (including glutathione). Although it was not the direct intention of the factorial model to determine absolute needs for each of the DAAs, it has been suggested that DAA intakes should be taken into consideration in defining the ideal composition of dietary protein to support muscle mass and health in both animals and humans [99]. In the context of the existing scientific literature, the findings of the factorial model warrant additional research to understand the role of and potential requirement for dietary DAAs.

The model predictions obtained for the MMD of protein and IAAs are subject to assumptions and limitations. A full discussion of these assumptions and limitations for the major sources of obligatory loss (oxidative losses [23] and endogenous gut losses [21]) has been provided previously. In relation to the oxidative losses, a more developed understanding has been obtained since our earlier publication [23], warranting an expanded discussion here. Specifically, our group has developed and has in press a mathematical model [the Nutritional Essential Amino Acid Demand model (NEAAD)] [100] describing the use of postabsorptive essential AA losses, extrapolated to 24 h, to garner a new perspective on essential AA requirements in healthy young humans consuming a habitual diet. In the NEAAD model, the rate of oxidative loss over the postabsorptive period (the model equivalent of PTO_PA_) is 22% of protein breakdown, whereas the present factorial model uses a rate of 11% to describe obligatory postabsorptive oxidative losses. The 2 models are conceptually different and based on different datasets with methodological differences, such as the amount of habitual dietary protein intake, which has been suggested to impact postabsorptive losses [25,101,102]. However, both datasets were developed from isotope tracer infusion studies, where a key limitation is the intracellular dilution of the tracer, which leads to an underestimation of the tracer enrichment and therefore the magnitude of any metabolic processes (such as oxidation) subsequently determined. A full explanation of the mechanisms and assumptions involved has been provided elsewhere [103]. It has also recently been suggested that established measures of whole-body protein breakdown may have been underestimated for a similar reason [25]. The data underpinning the IAA requirement estimates of the NEAAD model were specifically derived in a manner that accounts for intracellular dilution of the tracer [100]. The NEAAD model also used measurements of AA flux to estimate the composition of total body protein, in contrast to the AA composition values used in the present factorial model, which are equivalent to those underpinning current requirement estimates [17,31]. Although additional work is needed to understand the extent of oxidative IAA loss and protein breakdown in the postabsorptive state, it is worthwhile considering the implications of a potentially greater rate of oxidative loss (as PTO_PA_) for IAA and protein requirements. When a PTO_PA_ rate of 22% was incorporated into the model, both in isolation and combination with flux-based AA composition values, the resulting MMD for protein ranged from 646 to 873 mg/kgBW/d, remaining in line with current recommended intakes. For the IAAs, the MMD increased from as little as 5% (leucine) to as high as 91% (histidine), whereas scoring patterns (in mg/g protein intake) remained relatively constant with the original pattern determined for the factorial model (Table 4), with the exception of histidine. Importantly, regardless of the change applied, the IAA composition of total protein remained ∼50% higher than that of current requirement estimates (42% compared with 28%), emphasizing the need for experimental clarification. This further promulgated research, alongside that to obtain an accurate understanding of the magnitude of oxidative AA losses associated with postabsorptive protein breakdown, will contribute to ensuring that dietary recommendations are representative of true requirements. It is also worthwhile reiterating here that although a constant rate of IO was used in the factorial model (29% of an absorbed dietary AA), rates of IO across the AAs are likely to be nonconstant. For example, an IO rate of ≥50% for methionine was identified via direct measurements in rats [104], and amounts of splanchnic catabolism were found to differ between IAAs in the piglet [105]. Although the estimation of IO within the factorial model is reliant upon the existing data, which is limited to animal studies, further understanding in humans and regarding IAA-specific rates for IO is needed. As oxidation is the major source of obligatory AA loss contributing to dietary protein and IAA requirements, this understanding, in addition to that regarding postabsorptive oxidative AA losses, will increase the prediction capabilities of the model. An important consideration is the impact of gut endogenous losses, the second major source of AA loss, on gut microbial metabolism, as the composition and magnitude of these losses is expected to influence that of the microbial metabolites produced, and thus human health [106]. Although beyond the scope of the current model, the estimates produced here can be combined with models of microbial metabolism to predict how diets differing in protein quality and quantity affect this aspect of physiology. Although quantitatively minor, additional sources of loss play a role in overall protein and IAA requirements and are subject to limitations that represent potential sources of error. Primarily, data used for the estimation of losses because of UFAAP and HSNM were dated, with publication between 1948 and 1958. As the human diet and lifestyle has evolved substantially over the last 60 y, and both are known to affect the magnitude and/or AA composition of each of these sources of loss [2,33,107], the assumption that the values incorporated into the model reflect the current situation may be questionable. Although more recent human data for these losses were identified, they did not meet the constraints of the model. That is, they were not relevant to the healthy adult male and/or an energy-replete diet consisting of high-quality protein consumed at or near the current EAR for protein. Recent data meeting these constraints are needed to refine the model. Finally, although OL (because of functional roles and irreversible modification) represented the smallest relative loss for most AAs, with the least effect on MMD values in sensitivity analyses, these losses are complex and inherently difficult to quantify because of significant recycling within and communication across biochemical pathways [51,108]. For this reason, OL were limited to those representing the most established and well-researched pathways. Data representing losses because of functional roles of the SAAs were the most difficult to source and for the purposes of completeness, data from females as well as overweight but otherwise healthy individuals were included [52]. Although it has been proposed that excess bodyweight may create an abnormal pattern of SAA metabolism, removal of these data did not affect the value of OL for the SAAs. In addition, although a sex-specific influence on taurine excretion is apparent, males tend to have higher excretion rates compared with females [77]. Removal of data from mixed populations increased the magnitude of OL for SAA, indicating that the estimate of OL for SAA is conservative rather than excessive. Further research into the magnitude of functional AA losses is needed to better understand the relevance of these pathways for protein and IAA requirements. It is worth emphasizing that, as all losses have been modeled as minimum losses in a hypothetical diet containing the exact intakes of protein and IAAs to meet the requirements for the healthy adult male, the model requires adaptation for translation across different population groups. However, this requirement highlights a key strength of the model. Because each source of loss has been incorporated as a separate variable, different scenarios can be modeled and applied to create specific dietary protein demand estimates for key population groups as well as the individual. An additional and notable strength is incorporation of the most recent understanding of protein and AA metabolism, including mechanisms that have not necessarily been considered in previous methods implemented to develop protein and IAA requirement estimates.

In conclusion, a factorial model that allows the prediction of the MMD for protein and each IAA in the young adult male human has been developed. The model describes obligatory AA losses occurring after digestion and absorption in a hypothetical state where intake of total protein and each AA exactly meets requirements. Although not a direct reflection of the normal human diet, this approach allows the model to account for dietary protein consumption while not being subject to the influence of protein consumption above or below requirements on each source of loss, thus providing an elegant framework that can be further refined to account for different diets, populations, and physiological states. The model suggests that although the MMD for protein is similar to the current EAR, the total IAA demand is ∼40% greater than currently recommended, with the ideal ratio of IAAs to DAAs (41:59) close to that of whole-body protein and empirically derived optimal balances for animals. For some IAAs (tryptophan and threonine), the model predicts that the metabolic demand based on total obligatory loss over 24 h is ≥2-fold greater than currently recommended. Although subject to standard modeling limitations and assumptions, every care has been taken to ensure that the model and each component thereof reflects the most recent scientific understanding regarding protein and AA metabolism per the fed compared with fasted states. Subsequent iterations of the model will account for variations across demographics and life stages, such as adult females and older adults, as well the influence of protein and nonprotein dietary factors on requirements. The basal model can be further refined as additional data, such as differences in the rate of IO among AAs and across intake amounts, and more recent information regarding urinary AA and metabolite losses, become available.

## Author contributions

The authors’ responsibilities were as follows – CSS, PJM: were responsible for study design and modeling; CSS: was responsible for manuscript preparation, writing, and data/statistical analysis; CSS, PJM, RRW: were responsible for determining the final content of the manuscript; and all authors: read, reviewed, and approved the manuscript.

## Data availability

Data described in the manuscript will be made available upon request pending application and approval.

## Funding

This research was supported by the Riddet Institute, a New Zealand Centre of Research Excellence, funded by the Tertiary Education Commission of New Zealand.

## Conflict of interest

RRW is a shareholder in The Amino Company, holds United States patents for essential amino acid compositions, and has received grants from the National Cattleman’s Beef Association. The other authors report no conflicts of interest.
