# Supplementary material for: A Factorial Model of the Minimum Metabolic Demand for Protein and Indispensable Amino Acids in Young Adult Males: Implications for Current Recommendations
Source: J Nutr. 2026 Feb 12;156(4):101417. doi: 10.1016/j.tjnut.2026.101417 (PMC13084664; doi:10.1016/j.tjnut.2026.101417)
Supplement: multimedia component 1 [file mmc1.docx]

**SUPPLEMENTARY MATERIAL**

**Methods**

**Supplementary Table S1.** Data^1^ used to determine the mean AA composition of hair, skin, and nails for each AA.

| **Amino Acid** | **Lustig 1958 Data^1^** | | | | | |
| --- | --- | --- | --- | --- | --- | --- |
|  | **Epidermis** | **Skin** | **Nails** | **Hair 1** | **Hair 2** |  |
| IAA | g/100 g | | | | |  |
| Histidine | 3.22 | 2.03 | 2.72 | 2.01 | 3.19 |  |
| Isoleucine | 2.53 | 1.70 | 2.26 | 3.11 | 2.00 |  |
| Leucine | 5.26 | 3.14 | 5.06 | 4.15 | 3.86 |  |
| Lysine | 8.5 | 4.94 | 4.56 | 2.22 | 5.04 |  |
| Methionine | 0.58 | 0.59 | 0.58 | 0.43 | 0.4 |  |
| Cysteine | 2.55 | 0.5 | 7.67 | 12.8 | 12.2 |  |
| Phenylalanine | 1.54 | 1.32 | 1.54 | 1.22 | 1.38 |  |
| Tyrosine | 2.42 | 1.59 | 1.84 | 1.04 | 1.60 |  |
| Threonine | 3.57 | 2.62 | 4.40 | 6.10 | 5.88 |  |
| Tryptophan | 0.43 | 0.93 | 0.86 | 0.85 | 0.6 |  |
| Valine | 4.04 | 2.81 | 3.44 | 4.02 | 3.98 |  |
| DAA |  |  |  |  |  |  |
| Alanine | 2.96 | - | 3.64 | 2.68 | 3.43 |  |
| Arginine | 15.9 | 16.1 | 18.5 | 17.5 | 19.7 |  |
| Asp + Asn^2^ | 5.52 | 4.45 | 4.07 | 2.50 | 4.14 |  |
| Glu + Gln^2^ | 8.5 | 7.12 | 7.86 | 7.87 | 8.22 |  |
| Glycine | 13.3 | 15.9 | 4.14 | 4.68 | 4.77 |  |
| Proline | 2.52 | - | 2.52 | 3.18 | 5.78 |  |
| Serine | 14.12 | - | 5.73 | 8.62 | 9.73 |  |

^1^Data sourced from (1). ^2^No data for asparagine or glutamine were available; values represent aspartic acid and glutamic acid only. Asp, aspartic acid; Asn, asparagine; DAA, dispensable amino acids; Glu, glutamic acid; Gln, glutamine; IAA, indispensable amino acids; SD, standard deviation.

**Supplementary Table S2.** Data^1^ used for the calculation of UFAAP losses for each AA.

| **Paper** | | **Woodson 1948** | **Eckhardt 1948** | | | **Stein 1952** |
| --- | --- | --- | --- | --- | --- | --- |
|  |  |  | **Eckhardt 1948** | **Dunn 1947** | **Eckhardt unpublished** |  |
| Number of subjects | | 13 | 1 | 2 | 1 | 8 |
| Bodyweight (kg) | | 67 | 71 | 71 | 71 | 72.5 |
|  | | mg/24 hours | | | | |
| Histidine | Free | 188.3 | 120 |  | 159 |  |
|  | Total | 203.3 | 141 | 188.5 | 200 | 307 |
| Isoleucine | Free | 5.9 | 15.3 |  | 5.4 |  |
|  | Total | 20.3 | 23.3 | 19.3 | 17.7 | 24 |
| Leucine | Free | 9.6 | 12.1 |  | 16.2 |  |
|  | Total | 21.2 | 30.4 | 31.2 | 32.2 | 27 |
| Lysine | Free | 33.6 | 12 |  | 39.3 |  |
|  | Total | 73.2 | 75.3 | 83.1 | 120 | 61 |
| Methionine | Free | 7.8 | 4.4 |  | 4.9 |  |
|  | Total | 8.6 | 8.1 | 11.9 | 9.1 | 10 |
| Cysteine | Free | 87.7 |  |  |  |  |
|  | Total | 87.7 |  | 100 |  | 52 |
| Phenylalanine | Free | 16.4 | 15.5 |  | 24 |  |
|  | Total | 23.3 | 16.5 | 32.6 | 39.6 | 45 |
| Tyrosine | Free | 20.8 |  |  |  |  |
|  | Total | 52.5 |  |  |  | 76 |
| Threonine | Free | 24.4 | 19.5 |  | 24 |  |
|  | Total | 53.8 | 40.8 | 57.8 | 39.6 | 74 |
| Tryptophan | Free | 24.6 | 25.4 |  | 12.5 |  |
|  | Total | 41.4 | 33.3 |  |  |  |
| Valine | Free | 4.5 | 12.8 |  | 9.5 |  |
|  | Total | 19.8 | 24.4 |  | 29 | 30 |
| Alanine | Free |  |  |  |  |  |
|  | Total |  |  |  |  | 84 |
| Arginine | Free | 21.3 | 9.4 |  | 14 |  |
|  | Total | 23.7 | 30.3 | 35.6 | 39.3 | 20 |
| Asparagine | Free |  |  |  |  |  |
|  | Total |  |  |  |  | 54.5 |
| Aspartic acid | Free | 1.3 |  |  |  |  |
|  | Total | 164.5 |  | 190 |  | 212 |
| Glycine | Free |  |  |  |  |  |
|  | Total |  |  | 463 |  | 938 |
| Glutamic acid | Free | 35.8 |  |  |  |  |
|  | Total | 351.4 |  | 341.3 |  | 531 |
| Proline | Free | 8.5 |  |  |  |  |
|  | Total | 42.8 |  |  |  | 83 |
| Serine | Free |  |  |  |  |  |
|  | Total |  |  |  |  | 113 |

^1^Data sourced from (2-4) was presented as mg per 24 hours as both free amino acids and total (free plus bound as peptides or protein). Total data were used where possible. No data for glutamine were available. All measures represent the adult human male.

**Supplementary Table S3.** Data^1^ used to estimate losses due to the irreversible modification of histidine.

| **Reference** | | | **Urinary 3-methyl-histidine**  mg/24 hours |
| --- | --- | --- | --- |
| Soupart 1959 (5) | Evered | 33-47 | |
|  | Soupart | 35-87 | |
| Fuller and Garlick 1994 (6) | | 42.3 | |
| Long 1981 (7) | | 47.4 | |

^1^Data are specific to the adult human male.

**Supplementary Table S4.** Data^1^ used to estimate losses due to the irreversible modification of lysine.

| **Reference** | **Urinary**  **HLys + Gly-HLys** | **Units** |
| --- | --- | --- |
| Krane 1977 (8) | 0.216 | mol/mol HPro |
| Euli 1999 (9) | 100.41 | µmol/24 hours |
| Wheat 1989 (10) | 82.2 | µmol/24 hours |
| Rodriguez 1984 (11) | 38.625 | µmol/g Cr |
| Bisbee 1978 (12) | 60 | µmol/g Cr |
| Sato 1977 (13) | 110 | µmol/24 hours |
| Askenasi 1974 (14) | 52.2 | µmol/24 hours |

^1^Data are specific to the healthy adult human male in references (10, 11). In other references, the population was not specified, however (14) found no difference between sexes. HLys, hydroxylysine; Gly-HLys, glycosylated hydroxylysine; HPro, hydroxyproline

**Supplementary Table S5.** Data^1^ used to estimate losses due to functional roles of SAA.

| **Urinary metabolite** | **Olsen 2024**  **(15)** | **Olsen 2018**  **(16)** | **Soupart 1959**  **(5)** | **Turner 1964 (17)** | **Laksmanan 1976 (18)** |
| --- | --- | --- | --- | --- | --- |
|  | µmol/24 h | µmol/mmol creatinine | mg/24 hours | mmol/24 h | |
| Total homocysteine | 8.27 | 0.29 |  |  |  |
| Cystathionine | 36.6 | 0.182 |  |  |  |
| Taurine | 316 | 8.72 | 113 | 1.17 | 0.289 |
| Homolanthionine | 1.33 |  |  |  |  |
| Lanthionine | 14.3 |  |  |  |  |
| S-sulfocysteine | 6.53 |  |  |  |  |

^1^Data from (5, 17, 18) reflect values in healthy male adults. Data from (15, 16) reflect a mixed male and female population. These data were selected due to no other 24-hour data regarding non-taurine urinary sulphur metabolites being identified. SAA, sulphur amino acids (methionine and cysteine).

**Supplementary Table S6.** Data^1^ used to estimate losses due to functional roles of AAA.

| **Urinary metabolite** | **Mean** | **SD** |
| --- | --- | --- |
| Norepinephrine (µg/g creatinine) | 28.5 | 12.5 |
| Normetanephrine (µg/g creatinine) | 148.5 | 73 |
| VMA (mg/g creatinine) | 2.5 | 1 |
| Dopamine (µg/g) | 229 | 88.5 |
| HVA (mg/g) | 3.25 | 1.15 |
| Epinephrine (µg/g) | 6 | 3.5 |
| Metanephrine (µg/g) | 70.5 | 20 |

^1^Data for adult males from 15-25 years (19). HVA, homovanillic acid; VMA, vanillylmandelic acid.

**Supplementary Table S7.** Data^1^ used to estimate losses due to functional roles of tryptophan.

| **Reference** | **Urinary metabolite** | | | | |
| --- | --- | --- | --- | --- | --- |
|  | **5-HIAA** | **Kynurenine** | **KA** | **XA** | **6-SFT^2^** |
| Price 1956 (20) (mg/24 h) |  | 2.7 | 3 | 9.4 |  |
|  |  | 2.3 | 3.2 | 7 |  |
|  |  | 2.7 | 3 | 7.6 |  |
|  |  | 2.9 | 3 | 8.4 |  |
|  |  | 2.5 | 3 | 7.6 |  |
|  |  | 2.7 | 3 | 7.6 |  |
|  |  | 8.95 | 12.7 | 10.3 |  |
|  |  | 2.9 | 3.2 | 6.8 |  |
|  |  | 2.7 | 3.4 | 8 |  |
|  |  | 3.1 | 3.2 | 7.2 |  |
|  |  | 2.3 | 3.4 | 8 |  |
|  |  | 2.1 | 3 | 10.1 |  |
| Green 1980a (21) (mg/24 h) | 4.9 | 1.55 |  |  |  |
| Green 1980b (22) (mg/24 h) | 4.9 | 1.55 |  |  |  |
|  | 3.3 | 1.35 |  |  |  |
|  | 5.7 | 2.02 |  |  |  |
| Fideleff 2015 (23) (µg/24 h) |  |  |  |  | 4.3 |
|  |  |  |  |  | 6.6 |

^1^All data represent values obtained in the healthy male adult. ^2^Mean data were estimated using the published median value and the suggested formula for estimating the mean value from a small sample size (24). 5-HIAA, 5-hydroxyindoleacetic acid; 6-SFT, 6-sulfatoxymelatonin; KA, Kynurenic acid; XA, Xanthurenic acid. No data for anthranilic acid were available.

**Supplementary Table S8.** Data^1^ used to estimate glycine losses due to creatine metabolism.

| **Included study^1^** | **Creatinine** | | |
| --- | --- | --- | --- |
|  | g/day | g/kg/day | mgN/kg/day |
| Rand et al 1979 (25) | 1.59 | 0.026 | 9.46 |
|  | 1.46 | 0.024 | 8.94 |
|  | 1.78 | 0.024 | 8.85 |
|  | 1.73 | 0.019 | 7.09 |
|  | 1.59 | 0.024 | 8.79 |
| Komatsu et al 1983 (26) |  |  | 9.50 |
|  |  |  | 9.50 |
| Inoue et al 1973 (27) | 1.42 | 0.026 | 9.57 |
|  | 1.4 | 0.028 | 10.30 |
| Wayler et al 1983 (28) |  | 0.019 | 7.03 |
|  |  | 0.019 | 7.03 |
| Huang and Lin 1982 (29) | 1.15 | 0.019 | 7.20 |
| Todd et al 1984 (30) | 1.46 | 0.019 | 7.19 |

^1^Urinary creatinine data sourced from studies in young adult males consuming high quality protein at an intake meeting the current EAR for protein (25-28, 30). Urinary creatinine was reported in units of g/day and/or g/kg/day and/or mgN/kg/day.

**Supplementary Table S9.** Data^1^ used to estimate other losses of glycine and aspartate due to purine (uric acid excretion) and pyrimidine (beta-aminoisobutyrate excretion) metabolism, respectively.

| **Included study^1^** | **Subject bodyweight** | **Metabolite excretion** |
| --- | --- | --- |
| **Uric acid** | kg | g/day |
| Calloway 1975 | 71.5 | 0.364 |
|  |  | 0.384 |
| Puig 2012 | 65.6 | 0.52 |
| Waslien 1968 | 81 | 0.392 |
|  |  | 0.331 |
|  |  | 0.364 |
|  |  | 0.428 |
|  |  | 0.436 |
|  |  | 0.373 |
| **Beta-aminoisobutyrate** | | mmol/day (SD) |
| Nielsen & Killmann 1983 | 75^2^ | 0.15 (0.07) |

^1^Urinary uric acid data sourced from studies feeding high quality protein at an intake meeting or close to the current EAR for protein (31-33). An estimate for the urinary excretion of beta-aminoisobutyrate in healthy subjects was sourced from (34). While sex was not specified in this study, there is no apparent effect of sex on the urinary excretion of beta-aminoisobutyrate (35). SD, standard deviation

**Supplementary Table S10.** AA composition data^1^ and TID values^2^ used for calculation of DIAAS scores.

| **Protein source** | | **Indispensable Amino Acid** | | | | | | | | |
| --- | --- | --- | --- | --- | --- | --- | --- | --- | --- | --- |
|  |  | **Histidine** | **Isoleucine** | **Leucine** | **Lysine^3^** | **SAA** | **AAA** | **Threonine** | **Tryptophan^4^** | **Valine** |
|  |  | *mg/g protein* | | | | | | | | |
| **Milk** | |  | | | | | | | | |
| AA composition | Gorissen 2018 | 27 | 41 | 100 | 84 | 33 | 104 | 50 | 10* | 51 |
|  | Khan 2019 | 30 | 42 | 87 | 81 | 24 | 93 | 45 | 10 | 48 |
|  | Milan 2020 | 27 | 50 | 94 | 79 | 28 | 91 | 44 | 13 | 61 |
|  | Rutherfurd 1998 | 32 | 50 | 94 | 76 | 36 | 102 | 40 | 10 | 62 |
| TID (%) | Milk, liquid | 95.3 | 85.5 | 94.2 | 96.5 | 94.0 | 95.4 | 87.0 | 91.7 | 88.5 |
| **Whole egg** | |  |  |  |  |  |  |  |  |  |
| AA composition | Gorissen 2018 | 18 | 31 | 71 | 53 | 35 | 80 | 39 | 12* | 39 |
|  | Attia 2020 | 21 | 50 | 78 | 66 | 39 | 86 | 43 | 11 | 56 |
|  | Attia 2020 | 20 | 51 | 77 | 62 | 45 | 86 | 41 | 11 | 58 |
|  | Attia 2020 | 23 | 46 | 82 | 74 | 37 | 80 | 49 | 9 | 52 |
|  | Attia 2020 | 24 | 44 | 76 | 73 | 35 | 82 | 50 | 11 | 48 |
| TID (%) | Boiled egg | 85.1 | 92.0 | 92.8 | 94.9 | 95.2 | 93.1 | 87.4 | 87.5 | 91.4 |
| **Beef** | |  |  |  |  |  |  |  |  |  |
| AA composition | Mazhangara 2019 | 29 | 51 | 84 | 84 | 37 | 72 | 40 | 11 | 57 |
|  | Wu 2016 | 40 | 52 | 84 | 90 | 45 | 78 | 46 | 13 | 61 |
|  | Wu 2016 | 39 | 52 | 83 | 90 | 45 | 79 | 46 | 12 | 60 |
|  | Wu 2016 | 39 | 51 | 83 | 90 | 45 | 79 | 46 | 13 | 59 |
| TID (%) | Beef tenderloin | 98.7 | 99.0 | 99.5 | 99.1 | 99.0 | 98.7 | 98.4 | 99.5 | 99.6 |
| **Soy protein isolate** | |  |  |  |  |  |  |  |  |  |
| AA composition | Gorissen 2018 | 21 | 26 | 68 | 47 | 16 | 75 | 31 | 12* | 30 |
|  | Kalman 2014 | 26 | 48 | 77 | 60 | 25 | 89 | 36 | 13 | 47 |
|  | Vangsoe 2018 | 26 | 47 | 79 | 64 | 26 | 91 | 38 | 13 | 39 |
|  | Mohsen 2009 | 27 | 43 | 78 | 65 | 28 | 95 | 36 | 10 | 45 |
| TID (%) | SPI | 95.4 | 93.4 | 93.6 | 100 | 94.2 | 94.6 | 91.8 | 94.9 | 93.4 |
| **Rice** | |  |  |  |  |  |  |  |  |  |
| AA composition | Gorissen 2018 | 19 | 25 | 73 | 24 | 33 | 91 | 29 | 10* | 35 |
|  | Kalman 2014 | 23 | 44 | 83 | 30 | 53 | 112 | 39 | 15 | 57 |
|  | Joy 2013 | 22 | 41 | 80 | 31 | 49 | 101 | 35 | 14 | 58 |
| TID (%) | White rice | 93.3 | 91.7 | 89.8 | 100 | 79.7 | 90.7 | 94.1 | 94.0 | 91.5 |
| **Mung bean** | |  |  |  |  |  |  |  |  |  |
| AA composition | Yi-Shen 2018 | 28 | 39 | 74 | 62 | 13 | 90 | 28 | 6 | 46 |
|  | Dahiya 2013 | 32 | 43 | 76 | 65 | 15 | 81 | 32 | 12 | 51 |
| TID (%) | Whole mung bean | 81.4 | 80.7 | 83.6 | 81.8 | 81.5 | 86.0 | 77.4 | 79.8 | 80.4 |

^1^AA composition values were sourced from the following references, with first author and year noted in the table: milk (36-39); whole egg (36, 40); beef (41, 42); soy protein isolate (36, 43-45); rice (36, 43, 46); mung bean (47, 48). ^2^TID values were sourced from (49). ^3^TID values for lysine represent reactive lysine. ^4^Where tryptophan data were not reported (indicated with an asterisk*), tryptophan composition was sourced from the USDA food composition database, FoodData Central (50). AA, amino acid; IAA, indispensable amino acid; DIAAS, Digestible Indispensable Amino Acid Score; SPI, soy protein isolate; TID, true ileal digestibility; USDA, US Department of Agriculture.

**Results**

**Supplementary Table S11.** Sensitivity analysis^1^ showing the impact of change in each model parameter on the MMR for total protein and each AA.

| **Protein/AA** | **Magnitude of change in MMR per 1% change in model parameter^2^** | | | | | | |
| --- | --- | --- | --- | --- | --- | --- | --- |
|  | **IO** | **PTO_PP_** | **PTO_PA_** | **EGL** | **UFAAP** | **HSNM** | **OL** |
| Total protein | 1.839 | 0.622 | 1.551 | 1.456 | 0.359 | 0.313 | 0.201 |
| Histidine | 0.065 | 0.017 | 0.042 | 0.053 | 0.033 | 0.009 | 0.006 |
| Isoleucine | 0.062 | 0.022 | 0.054 | 0.064 | 0.003 | 0.009 | - |
| Leucine | 0.115 | 0.047 | 0.116 | 0.097 | 0.004 | 0.018 | - |
| Lysine | 0.107 | 0.045 | 0.113 | 0.071 | 0.010 | 0.020 | 0.002 |
| SAA | 0.076 | 0.022 | 0.054 | 0.064 | 0.012 | 0.021 | 0.013 |
| AAA | 0.121 | 0.045 | 0.113 | 0.107 | 0.013 | 0.015 | 0.001 |
| Threonine | 0.106 | 0.026 | 0.065 | 0.145 | 0.009 | 0.015 | - |
| Tryptophan | 0.022 | 0.007 | 0.019 | 0.015 | 0.006 | 0.003 | 0.003 |
| Valine | 0.086 | 0.030 | 0.076 | 0.088 | 0.003 | 0.014 | - |
| Total IAA | 0.761 | 0.262 | 0.653 | 0.701 | 0.094 | 0.124 | 0.026 |
| Alanine | 0.100 | 0.042 | 0.105 | 0.071 | 0.011 | 0.015 | - |
| Arginine | 0.105 | 0.045 | 0.112 | 0.050 | 0.004 | 0.048 | - |
| Asp + Asn | 0.159 | 0.053 | 0.132 | 0.150 | 0.034 | 0.019 | 0.003 |
| Glu + Gln | 0.220 | 0.076 | 0.191 | 0.166 | 0.074 | 0.032 | - |
| Glycine | 0.278 | 0.069 | 0.172 | 0.117 | 0.120 | 0.032 | 0.172 |
| Serine | 0.124 | 0.049 | 0.123 | 0.107 | 0.008 | 0.017 | - |
| Proline | 0.091 | 0.025 | 0.064 | 0.092 | 0.016 | 0.026 | - |
| Total DAA | 1.078 | 0.360 | 0.898 | 0.755 | 0.266 | 0.188 | 0.175 |

^1^The effect of variation in each parameter of the factorial model on the respective MMR was determined by altering the magnitude of each loss parameter independently and in 5% increments, up to a total variation range of *±*20%. ^2^The amount of change in the MMR per each 1% variation in a parameter is represented by the slope of the line when the MMR was then plotted as a function of variation for each parameter. AA, amino acid; IO, inevitable oxidation; PTO_PP_, oxidation associated with postprandial protein turnover; PTO_PA_, oxidation associated with postabsorptive protein turnover; EGL, gut endogenous losses; UFAAP, urinary free amino acids and peptides; HSNM, hair, skin, nails, and miscellaneous losses; OL, other losses; SAA, sulphur amino acids; AAA, aromatic amino acids; IAA, indispensable amino acids; DAA, dispensable amino acids; Asp, aspartic acid; Asn, asparagine; Glu, glutamic acid; Gln, glutamine.

**References**

1. Lustig B, Katchen B, Reiss F. The Amino Acid Composition of the Horny Layer of the Human Skin. Journal of Investigative Dermatology. 1958;30(3):159-63.

2. Eckhardt RD, Davidson CS. Urinary excretion of amino acids by a normal adult receiving diets of varied protein content. J Biol Chem. 1949;177(2):687-95.

3. Stein WH. A chromatographic investigation of the amino acid constituents of normal urine. J Biol Chem. 1953;201(1):45-58.

4. Woodson HW, Hier SW, et al. Urinary excretion of amino acids by human subjects on normal diets. J Biol Chem. 1948;172(2):613-8.

5. Soupart P. Urinary excretion of free amino acids in normal adult men and women. Clinica Chimica Acta. 1959;4(2):265-71.

6. Fuller MF, Garlick PJ. Human amino acid requirements: can the controversy be resolved? Annu Rev Nutr. 1994;14:217-41.

7. Long CL, Birkhahn RH, Geiger JW, Betts JE, Schiller WR, Blakemore WS. Urinary excretion of 3-methylhistidine: An assessment of muscle protein catabolism in adult normal subjects and during malnutrition, sepsis, and skeletal trauma. Metabolism. 1981;30(8):765-76.

8. Krane SM, Kantrowitz FG, Byrne M, Pinnell SR, Singer FR. Urinary excretion of hydroxylysine and its glycosides as an index of collagen degradation. J Clin Invest. 1977;59(5):819-27.

9. Euli D, Colombo L, Bruno A, Mussini E. Assay for 5-hydroxylysine and L-lysine in human and rat urine and in bone by gas chromatography. J Chromatogr B Biomed Sci Appl. 1999;724(2):373-9.

10. Wheat MR, McCoy SL, Barton ED, Starcher BM, Schwane JA. Hydroxylysine excretion does not indicate collagen damage with downhill running in young men. Int J Sports Med. 1989;10(3):155-60.

11. Rodriguez GP, Claus-Walker J. Measurement of hydroxylysine glycosides in urine and its application to spinal cord injury. J Chromatogr. 1984;308:65-73.

12. Bisbee WC, Kelleher PC. A method for measuring hydroxylysine and glycosylated hydroxylysines in urine and protein hydrolysates. Clin Chim Acta. 1978;90(1):29-36.

13. Sato T, Saito T, Kokubun M, Ito M, Yoshinaga K. Determination of hydroxylysine in urine. Tohoku J Exp Med. 1977;121(2):173-8.

14. Askenasi R. Urinary excretion of free hydroxylysine, peptide-bound hydroxylysine and hydroxylysyl glycosides in physiological conditions. Clin Chim Acta. 1975;59(1):87-92.

15. Olsen T, Vinknes KJ, Barvíková K, Stolt E, Lee-Ødegård S, Troensegaard H, et al. Dietary sulfur amino acid restriction in humans with overweight and obesity: Evidence of an altered plasma and urine sulfurome, and a novel metabolic signature that correlates with loss of fat mass and adipose tissue gene expression. Redox Biol. 2024;73:103192.

16. Olsen T, Øvrebø B, Turner C, Bastani NE, Refsum H, Vinknes KJ. Combining Dietary Sulfur Amino Acid Restriction with Polyunsaturated Fatty Acid Intake in Humans: A Randomized Controlled Pilot Trial. Nutrients. 2018;10(12).

17. Turner FP, Brum VC, Paquette WW, Welden RB. The urinary excretion of free taurine in acute and chronic disease, following surgical trauma, and in patients with acute alcoholism. Journal of Surgical Research. 1964;4(9):423-31.

18. Lakshmanan FL, Perera WD, Scrimshaw NS, Young VR. Plasma and urinary amino acids and selected sulfur metabolites in young men fed a diet devoid of methionine and cystine. The American Journal of Clinical Nutrition. 1976;29(12):1367-71.

19. Pussard E, Neveux M, Guigueno N. Reference intervals for urinary catecholamines and metabolites from birth to adulthood. Clinical Biochemistry. 2009;42(6):536-9.

20. Price JM, Brown RR, Ellis ME. Quantitative Studies on the Urinary Excretion of Tryptophan Metabolites by Humans Ingesting a Constant Diet1. The Journal of Nutrition. 1956;60(3):323-33.

21. Green AR, Aronson JK, Curzon G, Woods HF. Metabolism of an oral tryptophan load. I: Effects of dose and pretreatment with tryptophan. Br J Clin Pharmacol. 1980a;10(6):603-10.

22. Green AR, Aronson JK, Curzon G, Woods HF. Metabolism of an oral tryptophan load. II: Effect of pretreatment with the putative tryptophan pyrrolase inhibitors nicotinamide or allopurinol. Br J Clin Pharmacol. 1980b;10(6):611-5.

23. Fideleff HL, Fideleff G, Boquete HR, Suárez M, Azaretzky M. Male-female differences in 6-sulfatoxymelatonin excretion in hypopituitary patients. Arch Endocrinol Metab. 2016;60(3):223-30.

24. Hozo SP, Djulbegovic B, Hozo I. Estimating the mean and variance from the median, range, and the size of a sample. BMC Medical Research Methodology. 2005;5(1):13.

25. Rand WM, Scrimshaw NS, Young VR. An analysis of temporal patterns in urinary nitrogen excretion of young adults receiving constant diets at two nitrogen intakes for 8 to 11 weeks. Am J Clin Nutr. 1979;32(7):1408-14.

26. Komatsu T, Kishi K, Yamamoto T, Inoue G. Nitrogen requirement of amino acid mixture with maintenance energy in young men. J Nutr Sci Vitaminol (Tokyo). 1983;29(2):169-85.

27. Inoue G, Fujita Y, Niiyama Y. Studies on protein requirements of young men fed egg protein and rice protein with excess and maintenance energy intakes. J Nutr. 1973;103(12):1673-87.

28. Wayler A, Queiroz E, Scrimshaw NS, Steinke FH, Rand WM, Young VR. Nitrogen Balance Studies in Young Men to Assess the Protein Quality of an Isolated Soy Protein in Relation to Meat Proteins. The Journal of Nutrition. 1983;113(12):2485-91.

29. Huang PC, Lin CP. Protein requirements of young Chinese male adults on ordinary Chinese mixed diet and egg diet at ordinary levels of energy intake. J Nutr. 1982;112(5):897-907.

30. Todd KS, Butterfield GE, Calloway DH. Nitrogen balance in men with adequate and deficient energy intake at three levels of work. J Nutr. 1984;114(11):2107-18.

31. Calloway DH. Nitrogen Balance of Men with Marginal Intakes of Protein and Energy. The Journal of Nutrition. 1975;105(7):914-23.

32. Puig JG, Torres RJ, de Miguel E, Sánchez A, Bailén R, Banegas JR. Uric acid excretion in healthy subjects: a nomogram to assess the mechanisms underlying purine metabolic disorders. Metabolism. 2012;61(4):512-8.

33. Waslien CI, Calloway DH, Margen S. Uric Acid Production of Men Fed Graded Amounts of Egg Protein and Yeast Nucleic Acid123. The American Journal of Clinical Nutrition. 1968;21(9):892-7.

34. Nielsen HR, Killmann SA. Urinary excretion of beta-aminoisobutyrate and pseudouridine in acute and chronic myeloid leukemia. J Natl Cancer Inst. 1983;71(5):887-91.

35. Kvist E, Sjølin KE. Pseudouridine and beta-aminoisobutyric acid excretion in urine: diagnostic and prognostic value. Scand J Urol Nephrol. 1990;24(4):283-5.

36. Gorissen SHM, Crombag JJR, Senden JMG, Waterval WAH, Bierau J, Verdijk LB, et al. Protein content and amino acid composition of commercially available plant-based protein isolates. Amino Acids. 2018;50(12):1685-95.

37. Milan A, Samuelsson L, Shrestha A, Sharma P, Day L, Cameron-Smith D. Circulating Branched Chain Amino Acid Concentrations Are Higher in Dairy-Avoiding Females Following an Equal Volume of Sheep Milk Relative to Cow Milk: A Randomized Controlled Trial. Frontiers in Nutrition. 2020;7:553674.

38. Rutherfurd SM, Moughan PJ. The Digestible Amino Acid Composition of Several Milk Proteins: Application of a New Bioassay. Journal of Dairy Science. 1998;81(4):909-17.

39. Taj Khan I, Nadeem M, Imran M, Ullah R, Ajmal M, Jaspal M. Antioxidant properties of Milk and dairy products: A comprehensive review of the current knowledge. Lipids in Health and Disease. 2019;18.

40. Attia YA, Al-Harthi MA, Korish MA, Shiboob MH. Protein and Amino Acid Content in Four Brands of Commercial Table Eggs in Retail Markets in Relation to Human Requirements. Animals (Basel). 2020;10(3).

41. Mazhangara I, Chivandi E, Mupangwa JF, Muchenje V. The Potential of Goat Meat in the Red Meat Industry. Sustainability. 2019;11:3671.

42. Wu G, Cross HR, Gehring KB, Savell JW, Arnold AN, McNeill SH. Composition of free and peptide-bound amino acids in beef chuck, loin, and round cuts1,2. Journal of Animal Science. 2016;94(6):2603-13.

43. Kalman DS. Amino Acid Composition of an Organic Brown Rice Protein Concentrate and Isolate Compared to Soy and Whey Concentrates and Isolates. Foods. 2014;3(3):394-402.

44. Vangsoe MT, Thogersen R, Bertram HC, Heckmann LL, Hansen M. Ingestion of Insect Protein Isolate Enhances Blood Amino Acid Concentrations Similar to Soy Protein in A Human Trial. Nutrients. 2018;10(10).

45. Mohsen S, Fadel H, Bekhit M, Edris A, Ahmed M. Effect of substitution of soy protein isolate on aroma volatiles, chemical composition and sensory quality of wheat cookies. International Journal of Food Science & Technology. 2009;44:1705-12.

46. Joy J, Lowery R, Wilson J, Purpura M, De Souza E, Wilson S, et al. The effects of 8 weeks of whey or rice protein supplementation on body composition and exercise performance. Nutrition journal. 2013;12:86.

47. Dahiya P, Linnemann A, Boekel M, Khetarpaul N, Grewal R, Nout MJ. Mung Bean: Technological and Nutritional Potential. Critical reviews in food science and nutrition. 2013;55.

48. Yi-Shen Z, Shuai S, FitzGerald R. Mung bean proteins and peptides: nutritional, functional and bioactive properties. Food Nutr Res. 2018;62.

49. Hodgkinson SM, Stroebinger N, Stein HH, Fanelli N, de Vries S, van der Wielen N, et al. True ileal amino acid digestibility of human foods classified according to food type as determined in the growing pig. Journal of Nutrition. In press.

50. Agriculture UDo. FoodData Central: USDA; 2025 [Available from: <https://fdc.nal.usda.gov/>.
